# Repression of rRNA gene transcription by endothelial SPEN deficiency normalizes tumor vasculature via nucleolar stress

**DOI:** 10.1172/JCI159860

**Published:** 2023-10-16

**Authors:** Zi-Yan Yang, Xian-Chun Yan, Jia-Yu-Lin Zhang, Liang Liang, Chun-Chen Gao, Pei-Ran Zhang, Yuan Liu, Jia-Xing Sun, Bai Ruan, Juan-Li Duan, Ruo-Nan Wang, Xing-Xing Feng, Bo Che, Tian Xiao, Hua Han

**Affiliations:** State Key Laboratory of Cancer Biology, Department of Biochemistry and Molecular Biology, Fourth Military Medical University, Xi’an, China

**Keywords:** Vascular Biology, Cell stress, Endothelial cells

## Abstract

Human cancers induce a chaotic, dysfunctional vasculature that promotes tumor growth and blunts most current therapies; however, the mechanisms underlying the induction of a dysfunctional vasculature have been unclear. Here, we show that split end (SPEN), a transcription repressor, coordinates rRNA synthesis in endothelial cells (ECs) and is required for physiological and tumor angiogenesis. SPEN deficiency attenuated EC proliferation and blunted retinal angiogenesis, which was attributed to p53 activation. Furthermore, SPEN knockdown activated p53 by upregulating noncoding promoter RNA (pRNA), which represses rRNA transcription and triggers p53-mediated nucleolar stress. In human cancer biopsies, a low endothelial SPEN level correlated with extended overall survival. In mice, endothelial SPEN deficiency compromised rRNA expression and repressed tumor growth and metastasis by normalizing tumor vessels, and this was abrogated by p53 haploinsufficiency. rRNA gene transcription is driven by RNA polymerase I (RNPI). We found that CX-5461, an RNPI inhibitor, recapitulated the effect of *Spen* ablation on tumor vessel normalization and combining CX-5461 with cisplatin substantially improved the efficacy of treating tumors in mice. Together, these results demonstrate that SPEN is required for angiogenesis by repressing pRNA to enable rRNA gene transcription and ribosomal biogenesis and that RNPI represents a target for tumor vessel normalization therapy of cancer.

## Graphical abstract

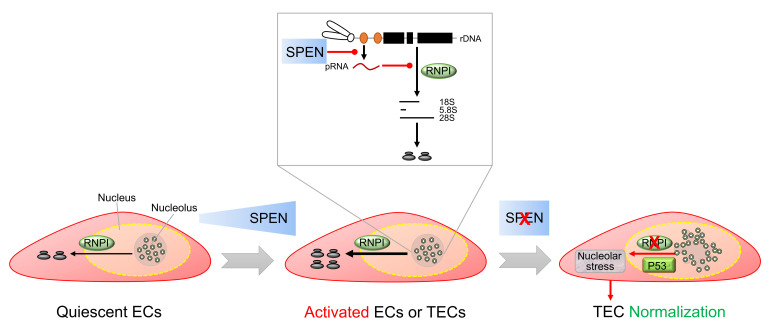

## Introduction

Angiogenesis, the growth of new vessels from existing ones, is required for vascularization of both physiological and pathological tissues (1). Tumor angiogenesis, however, is driven by abnormally high level of proangiogenic factors that thereby form a prosperous but chaotic vasculature characterized by disordered morphology, hyperactivated endothelial cells (ECs), and reduced pericyte and basement membrane coverage, leading to enhanced hypoxia and vessel leakage (2). The tumor vasculature promotes tumor growth and metastasis and blunts most current therapies. Antiangiogenesis therapy (AAT) normalizes chaotic tumor vessel structures and functions to compromise tumor malignancy and facilitate other therapies (3–5). To date, cytokines, signal transduction and gene expression regulators, and metabolic enzymes have been tested as AAT targets (3, 4, 6). However, the efficacy of current AATs has not been satisfactory in at least some cancers, and resistance often emerges early, prompting the discovery of new AAT targets (7, 8).

Recent single-cell RNA-Seq (scRNA-Seq) studies have revealed that ECs contain heterogeneous subsets with distinct proliferation, differentiation, and metabolic characteristics (9–11). Of note, compared with quiescent ECs, activated ECs, including tumor ECs (TECs), in angiogenesis exhibit higher expression of ribosome-related genes, implying that ribosome biogenesis is required for angiogenesis (9–11). Ribosomes are composed of ribosomal proteins (RPs) and rRNAs (12, 13). The 18S, 5.8S, and 28S rRNAs are encoded by ribosomal DNA (rDNA) and transcribed as a pre-rRNA by RNA polymerase I (RNPI) in nucleoli (14, 15). Extrinsic and intrinsic insults disrupting balanced ribosomal biogenesis interrupt the murine double minute (MDM) 2-p53 interaction, resulting in p53-mediated nucleolar stress, which is characterized by decreased ribosome biogenesis, deformed nucleolar morphology, and cell cycle arrest (16–18). As of yet, the role of ribosome biogenesis in angiogenesis remains unclear.

Split end (SPEN; also known as SMRT/HDAC1-associated repressor protein [SHARP] in humans and Msx2-interacting nuclear target protein [MINT] in mice) is an approximately 400 kDa large RNA-binding transcription repressor with a C-terminal SPEN paralog and ortholog C-terminal (SPOC) domain (19, 20). SPEN negatively regulates several signaling pathways, such as Notch (21, 22). SPEN also plays an essential role in X-chromosome inactivation by associating with X inactive specific transcript (XIST) and recruiting histone modification enzymes via the SPOC domain (23, 24). In addition, SPEN associates with the with the long noncoding RNA (lncRNA) steroid receptor RNA activator (SRA) that binds CCCTC-binding factor (CTCF) (20, 25) and participates in silencing endogenous retroviruses (26). SPEN-deficient mice are embryonic lethal and have multiple developmental disorders in their liver, pancreas, brain, and hematopoietic system (22), suggesting that SPEN plays a critical role in development. However, the role of SPEN in angiogenesis has not been elucidated. In this study, we demonstrate that SPEN is required for angiogenesis by enabling efficient rRNA transcription driven by RNPI. Endothelial SPEN deficiency, as well as the RNPI inhibitor CX-5461 (27–30), represses tumor growth via tumor vessel normalization. Therefore, the ribosome biogenesis machinery is a druggable target for AAT of human cancers.

## Results

### SPEN knockdown arrests EC proliferation and blunts angiogenesis.

**Figure 1:**
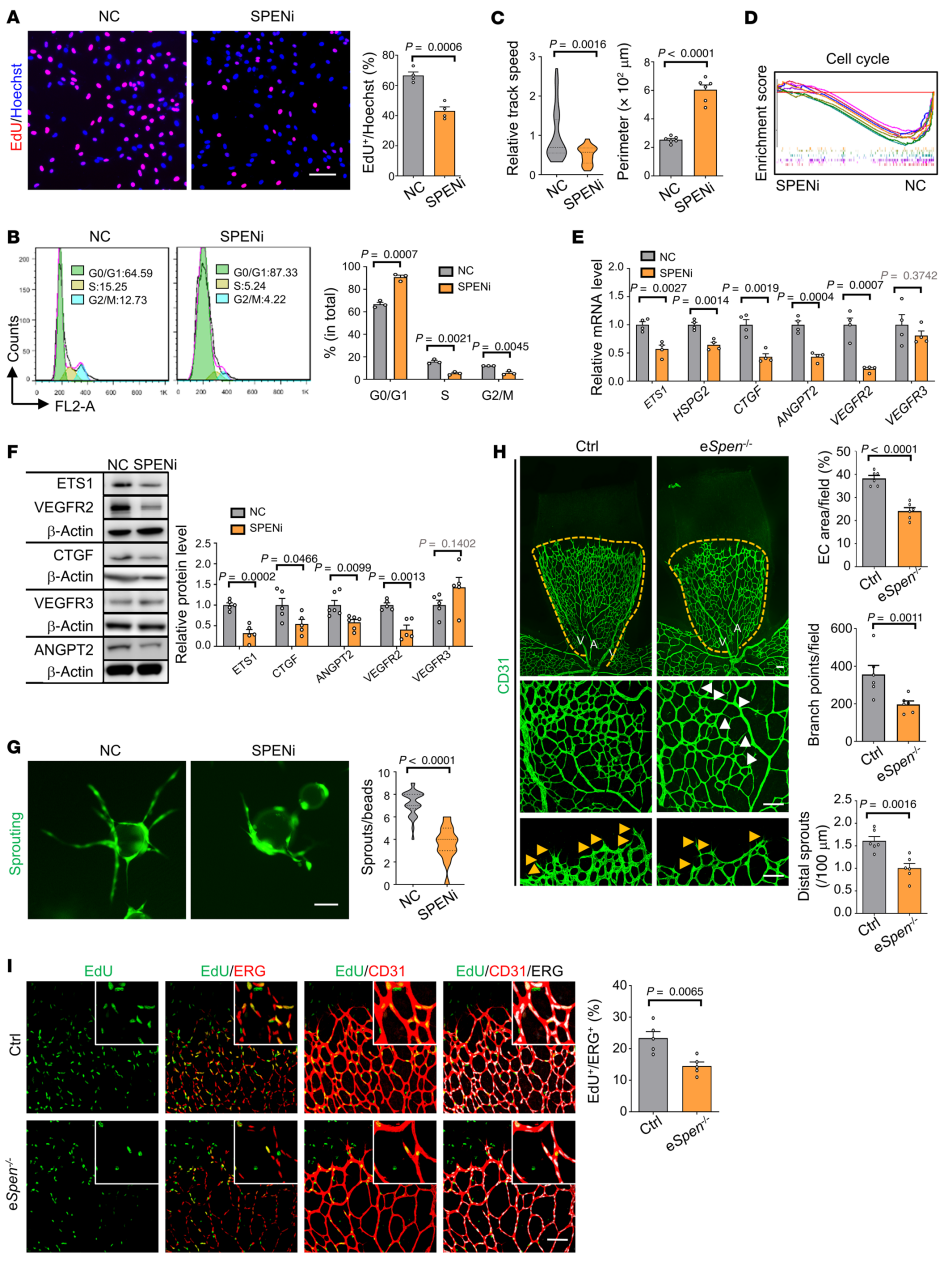
Endothelial SPEN deficiency represses EC proliferation and blunts angiogenesis. (**A–C**) HUVECs were transduced with NC or SPENi lentivirus expressing EGFP. Cell proliferation was determined by (**A**) EdU incorporation (*n* = 4) and (**B**) cell cycle analysis (*n* = 3). (**C**) ECs were recorded with a living-cell imaging workstation (Supplemental Figure 1F and Supplemental Videos, 1 and 2), and the relative track speeds of cells (*n* = 35 and 21 cell tracks for NC and SPENi, respectively) and cell perimeters (*n* = 6) were compared. Scale bar: 100 μm. (**D**) HUVECs were transduced with NC or SPENi lentivirus and subjected to RNA-Seq (*n* = 4). Cell cycle–related gene sets were analyzed by GSEA (color-coded gene sets are listed in Supplemental Figure 1H). (**E** and **F**) HUVECs were transduced with NC or SPENi lentivirus. The expression of angiogenesis-related genes was determined by (**E**) RT-qPCR (*n* = 4) and (**F**) immunoblotting (*n* = 5, except for *n* = 6 for ANGPT blots). β-Actin served as the loading control). (**G**) Sprouting was assessed by the microbead sprouting assay and quantitatively compared (*n* = 30 beads from 3 biological replicates). Scale bar: 100 μm. (**H**) The retinal vasculature of P6 pups was stained with anti-CD31 and photographed. The middle and bottom rows of images show the remodeling zone and angiogenic frontier of retinas, respectively. A, artery; V, vein; white arrows, vessel loops; yellow arrows, sprouts; yellow dashed lines, vascular radius. Scale bars: 100 μm. The EC area (*n* = 6), branch number (*n* = 6), and distal sprouts (*n* = 6) were quantified. (**I**) Immunostaining of mouse retinas after EdU labeling. EdU^+^ ECs were compared (*n* = 5). Scale bar: 100 μm. Data represent mean ± SEM. Unpaired 2-tailed Student’s *t* test was used.

Immunofluorescence of mouse tissue sections showed that SPEN is expressed in ECs (Supplemental Figure 1A; supplemental material available online with this article; https://doi.org/10.1172/JCI159860DS1). In human umbilical vein ECs (HUVECs), SPEN was detected exclusively in nuclei (Supplemental Figure 1B). To investigate the role of SPEN in ECs, we transduced HUVECs with lentivirus expressing a *SPEN* shRNA (shRNA2, referred to as SPENi hereafter; Supplemental Figure 1, C and D) or its nonsense control (NC). SPEN knockdown resulted in reduced proliferation of HUVECs, as shown by EdU incorporation and cell cycle analysis (Figure 1, A and B). Live-cell imaging showed that SPEN knockdown led to cell division arrest accompanied by enlarged cell size, and cell migration was mildly reduced (Figure 1C, Supplemental Videos 1 and 2, and Supplemental Figure 1, E and F). We performed RNA-Seq of HUVECs transduced with SPENi or NC lentivirus and analyzed data by principal component analysis (Supplemental Figure 1G). The result confirmed the downregulation of cell cycle–related genes in HUVECs with SPEN knockdown (Figure 1D and Supplemental Figure 1H). These data demonstrate that SPEN is required for EC proliferation.

Angiogenesis-associated genes, including ETS proto-oncogene 1 (*ETS1*), connective tissue growth factor (*CTGF*), angiopoietin 2 (*ANGPT2*), *VEGFR2*, and heparan sulfate proteoglycan 2 (*HSPG2*), were downregulated in HUVECs with SPEN knockdown, as confirmed by quantitative reverse transcription PCR (RT-qPCR), immunoblotting, and immunofluorescence (Figure 1, E and F, and Supplemental Figure 1, I and J). Consistently, the in vitro sprouting assay showed that SPEN knockdown compromised HUVEC sprouting (Figure 1G). To determine the role of SPEN in angiogenesis in vivo, we induced EC-specific *Spen* ablation in *Cdh5-Cre^ERT2^-Spen^fl/fl^* (e*Spen^–/–^*) P1 pups and adult mice using tamoxifen (Supplemental Figure 2, A–E) (31). On P6, retinal whole-mount CD31 staining showed that while the radius of the retinal vasculature did not change, EC areas, vessel branch points, and distal vessel sprouts decreased, with some microvessels appearing “broken” in e*Spen^–/–^* pups (Figure 1H). A Matrigel plug assay showed that, while the Matrigel plugs were well vascularized on day 7 in the control mice, vascularization was almost blocked in e*Spen^–/–^* mice (Supplemental Figure 2F). EdU incorporation and Ki67 staining showed that *Spen* ablation resulted in reduced EC proliferation (Figure 1I and Supplemental Figure 2, G and H). Together, these results indicate that endothelial SPEN is required for angiogenesis by supporting EC proliferation.

### SPEN knockdown arrests EC proliferation via p53.

**Figure 2:**
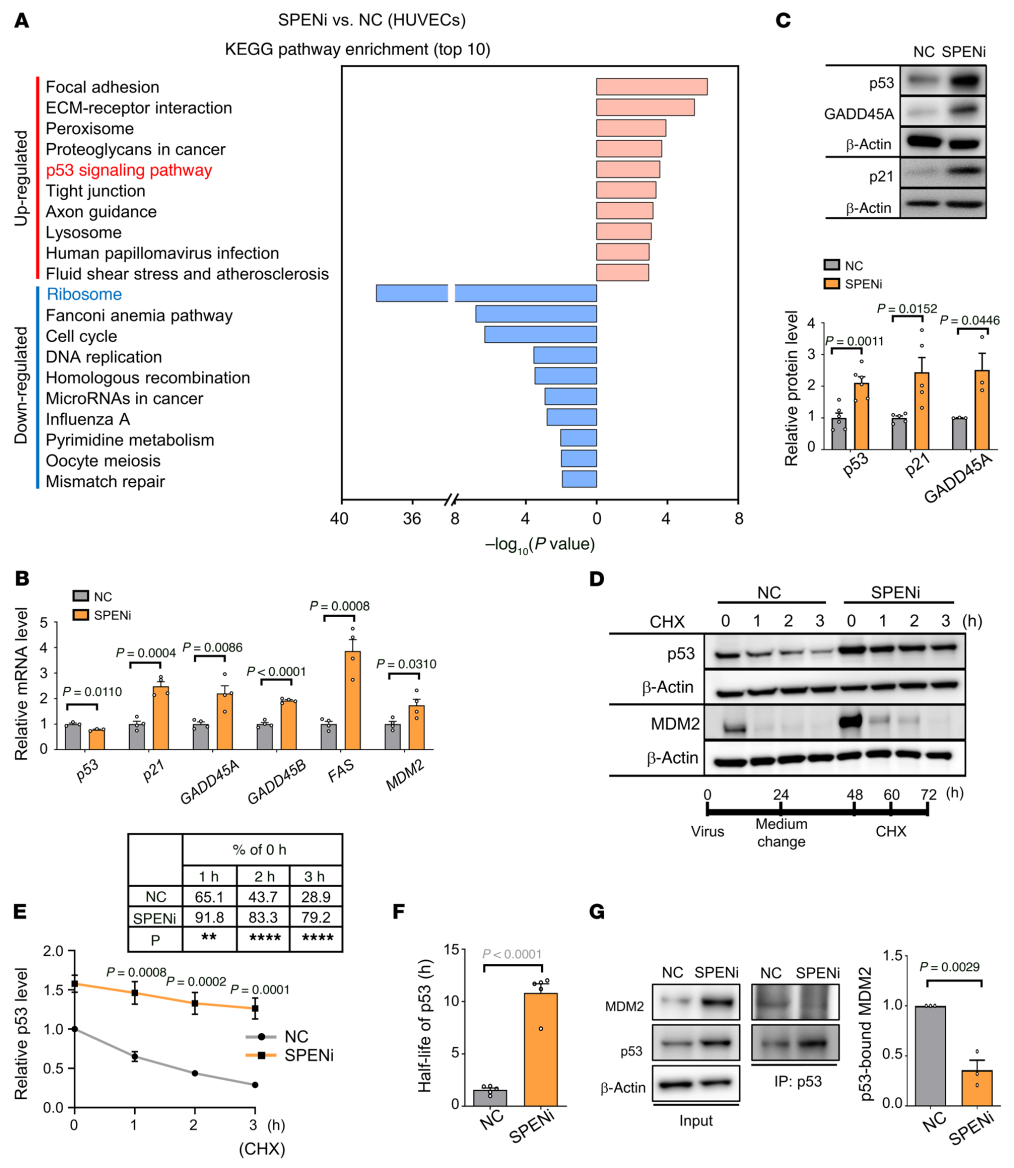
SPEN knockdown activates p53. (**A**) Signature genes that are differentially expressed in HUVECs transduced with NC or SPENi lentivirus; the top 10 markedly changed entries were presented. (**B** and **C**) HUVECs were transduced with NC or SPENi lentivirus. The expression of p53 and its downstream genes was determined by (**B**) RT-qPCR (*n* = 4, except for n = 3 in *p53*) and (**C**) immunoblotting (*n =* 6, 5, and 3 for p53, p21, and GADD45A, respectively). β-Actin served as the loading control. (**D**–**F**) HUVECs were transduced with NC or SPENi lentivirus and cultured with cycloheximide (CHX) as depicted. The p53 and MDM2 levels were assessed by immunoblotting at 0, 1, 2 and 3 hours after CHX addition (*n* = 5). β-Actin served as the loading control. The (**E**) p53 level and its (**F**) half-life were determined. The table in **E** shows the percentage of p53 level at different time points versus p53 level at 0 hours after CHX addition (**P < 0.01; ****P < 0.0001). (**G**) HUVECs were transduced with NC or SPENi lentivirus. Cell extracts were precipitated with anti-p53 and immunoblotted with anti-MDM2 (*n* = 3). Data represent mean ± SEM. Unpaired 2-tailed Student’s *t* test was used.

Gene expression profiling revealed that p53 signaling was remarkably upregulated in HUVECs with SPEN knockdown (Figure 2A and Supplemental Figure 3A) (32). RT-qPCR and immunoblotting confirmed that p53 downstream molecules, including *p21* and growth arrest and DNA damage inducible α (*GADD45A*), were upregulated in HUVECs with SPEN knockdown, while p53 was upregulated at the protein but not the mRNA level (Figure 2, B and C). Further analyses showed that nuclear p53 increased accompanied by increased transactivation activity, as shown by immunoblotting and reporter assay, respectively (Supplemental Figure 3, B and C). The p53 protein level is predominantly regulated by MDM2, which prevents proteasome-mediated p53 degradation via protein-protein interaction (32, 33). We treated HUVECs transduced with SPENi or NC with cycloheximide and monitored p53 level by immunoblotting. The result showed that SPEN knockdown delayed the decrease of p53 and prolonged its half-life, suggesting inhibited degradation, while MDM2 degradation appeared unaltered (Figure 2, D–F, and Supplemental Figure 3D). Consistently, p53-associated MDM2 decreased in SPENi-transfected HUVECs, as determined by immunoprecipitation (Figure 2G). These results suggest that SPEN knockdown in ECs results in p53 activation by delayed degradation.

**Figure 3:**
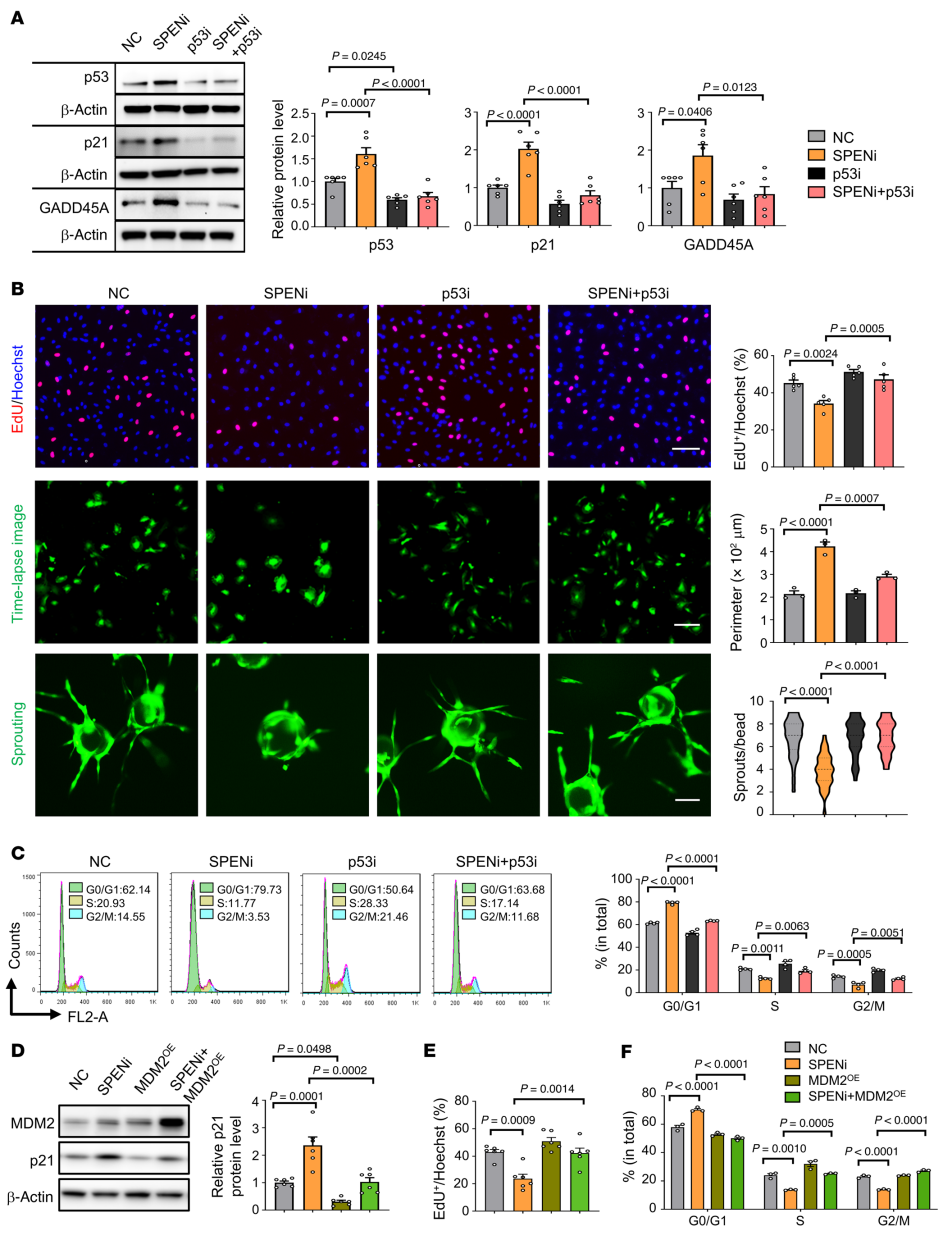
SPEN knockdown represses EC proliferation by activating p53. (**A**–**C**) HUVECs were transduced with NC, SPENi, p53i, or SPENi+p53i lentivirus expressing EGFP. (**A**) The expression of p53, p21, and GADD45A was determined by immunoblotting (*n* = 6). β-Actin served as the loading control. (**B**) The cell proliferation (*n* = 5), cell perimeter (*n* = 3), and sprouts (*n* = 30 beads from 3 biological replicates) were determined by the EdU incorporation, live-cell imaging, and microbead sprouting assay, respectively. Scale bars: 100 μm. (**C**) The cell cycle progression was determined by FACS (*n* = 4). (**D**–**F**) HUVECs were transduced with SPENi or NC and simultaneously transduced with MDM2-overexpressing lentivirus. The (**D**) p21 expression, (**E**) cell proliferation, and (**F**) cell cycle progression were determined (*n* = 6 for **D** and **E**, *n* = 3 for **F**). Data represent mean ± SEM. One-way ANOVA with Tukey’s multiple comparisons test was used.

To assess the role of p53 in the SPEN knockdown–induced proliferation arrest in ECs, we transduced HUVECs with SPENi together with a lentivirus expressing *p53* shRNA (p53i). p53 knockdown abrogated the upregulation of p21 and GADD45A in HUVECs transfected with SPENi (Figure 3A). Consequently, p53 knockdown ameliorated the SPENi-induced proliferation arrest, cell size enlargement, and sprouting defects, as determined by EdU incorporation, live-cell imaging, and sprouting assay, respectively (Figure 3B). Cell cycle analysis confirmed that p53 knockdown rescued SPENi-induced G_1_ arrest (Figure 3C). Knockdown of p21 with shRNA (p21i) showed similar effects (Supplemental Figure 3, E–G). We also transduced HUVECs with SPENi or NC and a MDM2 overexpression lentivirus simultaneously. The result showed that overexpressing MDM2 rescued p21 expression and cell proliferation (Figure 3, D–F, and Supplemental Figure 3, H–J). These results indicate that SPEN knockdown represses EC proliferation by activating p53.

### SPEN knockdown upregulates pRNA to thereby downregulate pre-rRNA transcription, leading to nucleolar stress activation of p53.

Next, we set out to determine the mechanism underlying p53 activation in ECs with SPEN knockdown. We examined Ser15, Ser20, and Thr18 phosphorylation of p53, which is involved in p53-MDM2 interaction and activation (34, 35), by immunoblotting. The result showed that SPEN knockdown did not change p53 phosphorylation at these residues remarkably (Supplemental Figure 3K). PIN1, a peptidyl-prolyl cis-trans isomerase binding to Thr81-phosphorylated p53 to thereby prevent p53-MDM2 interaction (36, 37), was downregulated in HUVECs with SPEN knockdown (Supplemental Figure 3L). Therefore, p53 upregulation might not be resulted from altered phosphorylation.

**Figure 4:**
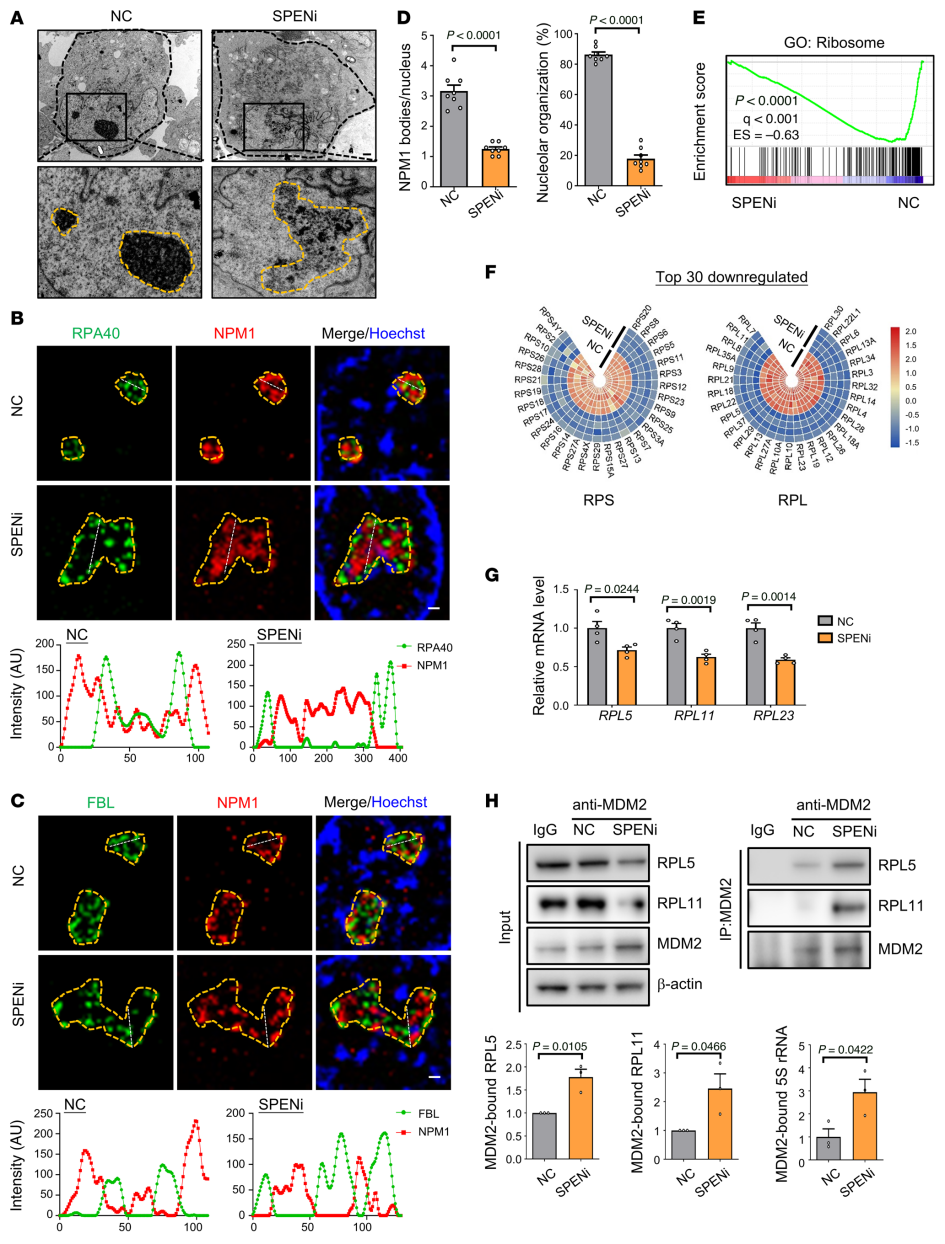
SPEN knockdown triggers nucleolar stress. (**A**) HUVECs were transduced with NC or SPENi lentivirus and observed under TEM. Dashed yellow lines indicate nucleoli. Scale bar: 1 μm. (**B**–**D**) HUVECs were transduced with NC or SPENi lentivirus and subjected to immunostaining followed by SIM microscopy. Dashed yellow lines indicate nucleoli. The NPM1, UBF, and RPA40 intensities along the white lines are plotted. Scale bar: 1 μm. (**D**) The NPM1 body number and HUVECs with normal nucleoli were counted (*n* = 8). (**E** and **F**) Analyses of ribosome-related genes by (**E**) GSEA and (**F**) heatmaps in HUVECs transduced with NC or SPENi lentivirus followed by RNA-Seq. (**G**) HUVECs were transduced with NC or SPENi lentivirus. The expression of *RPL5*, *RPL11*, and *RPL23* was determined by RT-qPCR (*n* = 4). (**H**) HUVECs were transduced with NC or SPENi lentivirus. Cell lysates were immunoprecipitated with anti-MDM2 after ultracentrifugation and detected with anti-RPL5 and RPL11 or RT-qPCR for 5S rRNA (*n* = 3). Data represent mean ± SEM. Unpaired 2-tailed Student’s *t* test was used.

RNA-Seq showed that the ribosome-related genes were downregulated in HUVECs with SPEN knockdown (Figure 2A). Ribosomes are generated in nucleoli, which are phase-separated, membrane-less organelles with a fibrillar center (FC) surrounded by a dense fibrillar component (DFC) layer and an outside granular component (GC) layer (16, 17, 38). Disturbed ribosomal biogenesis leads to p53 activation by disrupting the MDM2-p53 interaction, a process called nucleolar stress (16–18). In HUVECs, SPEN knockdown resulted in irregularly shaped nucleoli that unraveled throughout the nucleoplasm in dispersed fibrillar structures, in contrast to round and regular nucleoli in the controls (Figure 4A). Immunostaining of the nucleolar markers RPA40 (FC), FBL (DFC), and NPM1 (GC) followed by structured illumination microscopy (SIM) confirmed that although SPEN did not appear in nucleoli, SPEN knockdown resulted in the fusion of nucleoli, where the FC and DFC regions relocated to the nucleolar periphery and surrounded the remnant GC, appearing as unraveled “nucleolar necklaces” (Figure 4, B and C, and Supplemental Figure 4A) (17). Quantitative analyses showed that, in contrast to that in controls, the FC and DFC markers were distributed outside the GC area in SPEN-knockdown HUVECs, leading to deformed nucleoli (Figure 4, B–D). Functionally, RP gene expression was downregulated in HUVECs with SPEN knockdown (Figure 4, E–G). Moreover, in SPENi-transfected HUVECs, MDM2-bound RPL5 and RPL11, as well as 5S rRNA, increased markedly, suggesting that SPEN knockdown increased MDM2 associated with RPs in the form of ribonucleoprotein particle containing RPL5, RPL11, and 5S rRNA (Figure 4H) (16–18). These results demonstrate that SPEN is required for maintaining the nucleolar structure and function in ECs and SPEN deficiency triggers nucleolar stress to thereby activate p53 in ECs.

**Figure 5:**
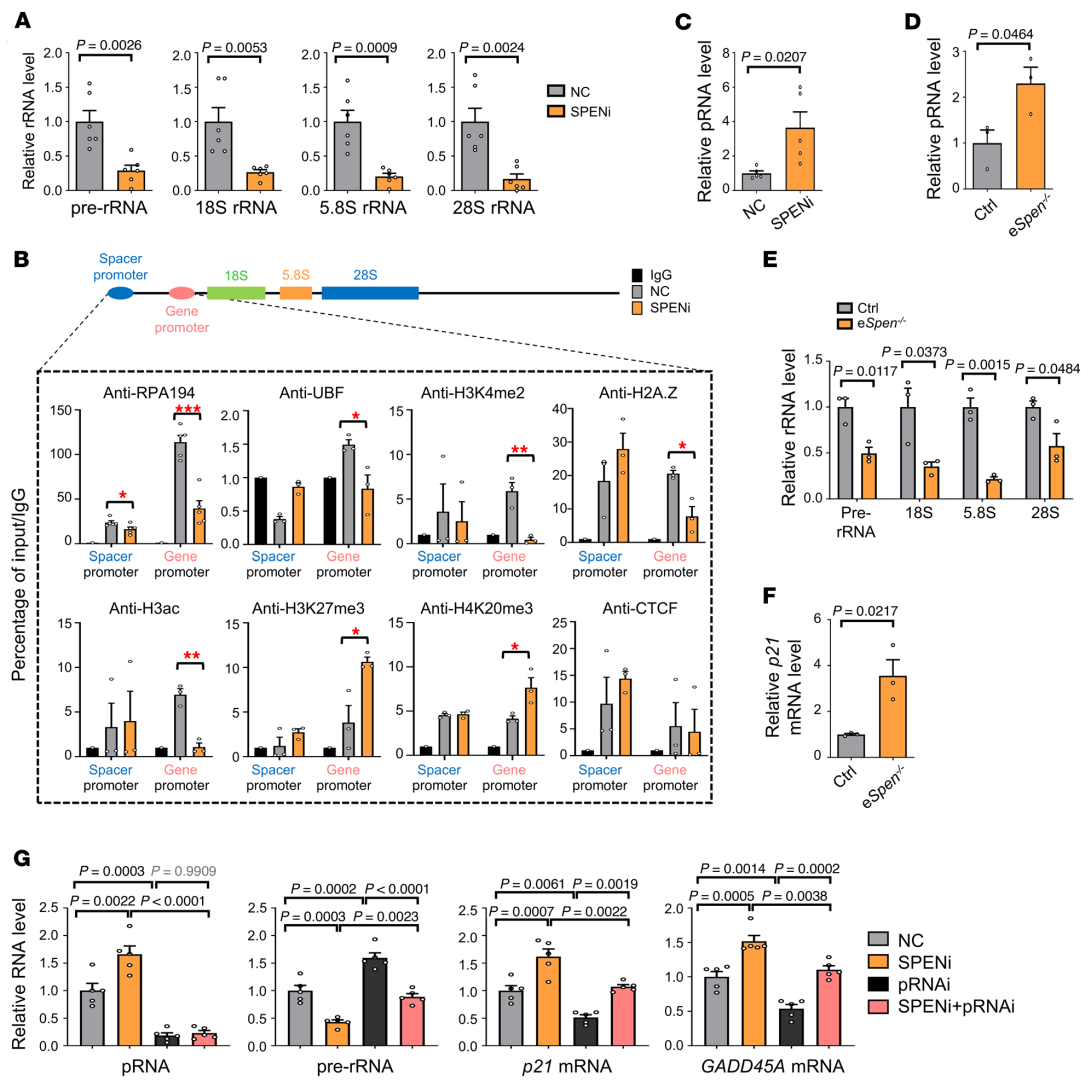
SPEN knockdown triggers nucleolar stress by upregulating pRNA in ECs. (**A**) HUVECs were transduced with NC or SPENi lentivirus. The expression of pre-rRNA, 18S, 5.8S, and 28S rRNA was determined by RT-qPCR (*n* = 6). (**B**) HUVECs were transduced with NC or SPENi lentivirus. ChIP-qPCR was performed with anti-RPA194, anti-UBF, anti-H3K4me2, anti-H2A.Z, anti-H3ac, anti-H3K27me3, anti-H4K20me3, and anti-CTCF antibodies (*n* = 3, except for *n* = 5 in anti-RPA194). *P < 0.05; **P < 0.01; ***P < 0.001. (**C**) HUVECs were transduced with NC or SPENi lentivirus. pRNA expression was determined by strand-specific RT-qPCR (*n* = 5). (**D**–**F**) ECs from P6 retinas of e*Spen^–/–^* and control mice were analyzed by RT-qPCR for (**D**) the expression of pRNA, (**E**) pre-rRNA and mature rRNAs, and (**F**) *p21* (*n* = 3, each sample is a pool of 3–4 retinas). (**G**) HUVECs were transduced with lentivirus as indicated. The expression of pRNA, pre-rRNA, *p21*, and *GADD45A* was determined by RT-qPCR (*n* = 5). Data represent mean ± SEM; 1-way ANOVA with Tukey’s multiple comparisons test was used in **G**; unpaired 2-tailed Student’s *t* test was used for **A**–**F**.

Furthermore, we explored how SPEN deficiency impaired ribosome biogenesis in ECs. NPM1 sustains nucleolar organization (39). However, the NPM1 protein level was not influenced by SPEN knockdown (Supplemental Figure 4B). In humans and mice, approximately 300 rDNA copies per haploid genome are arranged as tandem repeats on the short arms of acrocentric chromosomes (14, 15, 38). Each rDNA unit is divided into an approximately 13 kb pre-rRNA–encoding gene and an approximately 30 kb intergenic spacer (IGS) region, which contains an rRNA gene promoter proximal to and responsible for pre-rRNA gene transcription and an upstream spacer promoter that enhances the gene promoter (14, 15, 40–42). We found that pre-rRNA and processed 18S, 5.8S, and 28S rRNAs were downregulated in SPENi-transfected HUVECs (Figure 5A), suggesting that SPEN knockdown downregulates rRNA gene expression, leading to nucleolar stress (16–18).

RNPI-mediated rDNA transcription is controlled at several levels. Histone modifications, histone exchange, and the upstream binding factor–mediated (UBF-mediated) nucleosome replacement epigenetically regulate rDNA, while preinitiation complex assembly requires CTCF, DNA isomerases, and cohesin/condensin complexes (14, 15, 43–45). Moreover, at least 3 noncoding RNAs (ncRNAs) regulate rRNA gene transcription (Supplemental Figure 4C) (46–50): RNPI-driven promoter RNA (pRNA) silences pre-rRNA genes on inactive rDNA loci; stress-induced promoter and pre-rRNA antisense RNA (PAPAS) is transcribed by RNPII from the IGS in the antisense direction and inhibits pre-rRNA gene transcription; and IGS-derived sense and antisense ncRNAs regulate rRNA transcription via R-loop formation. We examined RNPI and UBF uploading as well as histone modifications around the promoter region of the rDNA repeats by ChIP–quantitative PCR (ChIP-qPCR) (44). RNPI and UBF binding was markedly decreased around the gene promoter of rDNA repeats, accompanied by decreased activation (H3K4me2, H2A.Z, H3ac) and increased repression histone marks (H3K27me3, H4K20me3) (Figure 5B). RNPI recruitment is dependent on CTCF, which associates with lncRNA SRA that binds SPEN (20, 25). However, the binding of CTCF to rDNA was not changed (Figure 5B), and in our view, overexpressing CTCF did not upregulate pre-rRNA in HUVECs (Supplemental Figure 4D). Next, by using strand-specific RT-qPCR, we found that the IGS transcripts from both sense and antisense chains were not altered after SPENi transfection (Supplemental Figure 4E). Similarly, the PAPAS level was comparable between SPENi- and NC-transfected HUVECs (Supplemental Figure 4F). Finally, chain-specific RT-qPCR showed that the pRNA level increased substantially in SPENi-transfected HUVECs (Figure 5C), suggesting that SPEN knockdown in ECs results in disrupted nucleolar structure and functions likely via the upregulated pRNA.

We also evaluated *Spen* knockout–induced nucleolar stress in vivo. Immunofluorescence detected deformed nucleoli in ECs in the angiogenic retinas of e*Spen*^–/–^ pups, accompanied by upregulated pRNA, downregulated pre-rRNA and mature rRNA, and upregulated *p21* in retinal ECs (Figure 5, D–F, and Supplemental Figure 4G). However, we did not detect altered expression of pRNA, rRNA, and *p21* in adult brain ECs from e*Spen^–/–^* mice (Supplemental Figure 4H), suggesting that SPEN is not required for maintaining nucleoli in quiescent ECs.

To determine whether pRNA upregulation is responsible for pre-rRNA downregulation and p53 activation in HUVECs with SPEN knockdown, we transfected HUVECs with SPENi together with a pRNA antisense oligonucleotides (ASOs) to knockdown pRNA. The result showed that pRNA knockdown completely rescued the pre-rRNA expression and abrogated the SPENi-induced upregulation of *p21* and *GADD45A* (Figure 5G). Consistently, a time course observation showed that the pre-rRNA downregulation preceded the *p21* and *GADD45A* upregulation in HUVECs with SPEN knockdown, and p53 or p21 knockdown failed to rescue SPENi-induced nucleolar deformation, suggesting that SPEN knockdown activates p53 after reducing pre-rRNA transcription (Supplemental Figure 4, I–K). Together, these results demonstrate that SPEN knockdown upregulates pRNA to attenuate rRNA transcription, leading to nucleolar stress and p53 activation in ECs.

To further examine the role of SPEN in ECs, we tried to upregulate SPEN in HUVECs using CRISPR-mediated activation of the SPEN promoter. Three sgRNAs were designed, and SPEN^OE3^ (referred to as SPEN^OE^ hereafter) exhibited highest SPEN upregulation, as confirmed by RT-qPCR and immunofluorescence (Supplemental Figure 5, A and B). RT-qPCR showed that the pRNA level decreased, while the pre-rRNA and 5S rRNA levels were upregulated, but 18S and 28S rRNA did not change significantly (Supplemental Figure 5, C and D). The p53 level and those of its downstream molecules, p21 and GADD45A, were reduced (Supplemental Figure 5E). Cell proliferation increased mildly, as shown by the cell cycle analysis, accompanied by enhanced sprouting ability (Supplemental Figure 5, F and G). These results further indicate that SPEN represses pRNA to facilitate rRNA transcription and EC proliferation.

### Endothelial Spen ablation inhibits tumor growth.

**Figure 6:**
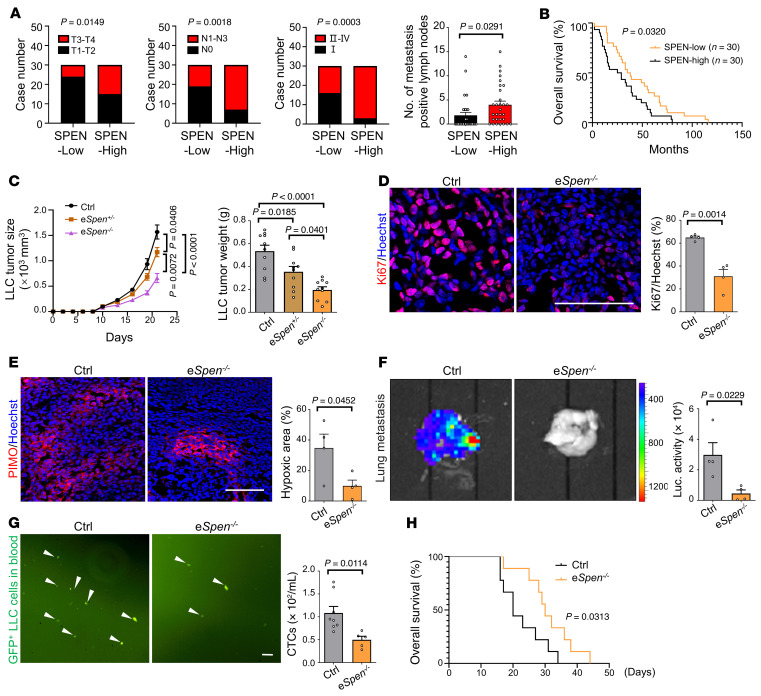
Endothelial *Spen* ablation represses tumor growth. (**A** and **B**) Human lung cancer biopsies were immunostained for CD31 and SPEN, and the SPEN intensity in the CD31^+^ area was quantified. (**A**) Tumor progression was analyzed between the endothelial SPEN–high and SPEN–low groups. (**B**) The correlation of endothelial SPEN level with overall survival was evaluated by Kaplan-Meier analysis. *n* = 30 patients per group. (**C**) Mice were inoculated with LLC cells. Tumor size was monitored, and tumor weights were compared on day 21 after inoculation (*n* = 10). (**D** and **E**) Control and e*Spen^–/–^* mice were inoculated with LLC cells. (**D**) Tumors at day 21 after inoculation were immunostained with Ki67 and quantitatively compared (*n* = 4). Scale bar: 100 μm. (**E**) Tumor hypoxia was evaluated by staining with Hypoxyprobe (*n* = 4). (**F**) Mice were inoculated with luciferase^+^ LLC cells. Tumors were removed on day 14 after inoculation, and the mice were maintained for an additional 28 days. Lung metastasis was evaluated using chemoluminescence (*n* = 4). (**G**) Mice were inoculated with GFP^+^ LLC cells. Circulating GFP^+^ LLC cells in blood were counted on day 21 after inoculation (*n* = 8 and 5 for control and e*Spen^–/–^*, respectively). The white arrowheads denote GFP^+^ LLC cells in blood. Scale bar: 100 μm. (**H**) Mice were inoculated with LLC cells. Tumors were removed on day 21 after inoculation, and mouse survival was plotted thereafter (*n* = 9). Data represent mean ± SEM. Log-rank (Mantel-Cox) test was used for **B** and **H**; 1-way ANOVA with Tukey’s multiple comparisons test was used for **C**; χ^2^ analyses were used for **A**, except for no. of metastasis positive lymph nodes; unpaired 2-tailed Student’s *t* test was used for **D**–**G** and no. of metastasis positive lymph nodes in **A**.

TECs exhibit higher expression of ribosome-related genes (9–11). In human lung cancer biopsies, immunostaining showed that lower endothelial SPEN level correlated with lower TNM and AJCC stages and higher endothelial SPEN level correlated with more lymph node metastasis (Figure 6A and Supplemental Figure 6A). Moreover, a low endothelial SPEN level correlated with extended patient overall survival (Figure 6B). Consistently, in gastric cancer and breast cancer, lower endothelial SPEN expression correlated with extended patient survival (Supplemental Figure 6, B and C). In TECs from Lewis lung carcinoma–bearing (LLC-bearing) mice, the *Spen* mRNA level increased along with tumor progression (Supplemental Figure 6D). Therefore, high endothelial SPEN level positively correlates with tumor progression in both human and mouse models.

Then, we inoculated SPEN-deficient and control mice with LLC or B16-F10 melanoma cells (Supplemental Figure 2B and Supplemental Figure 6E). Tumor growth was retarded in endothelial SPEN–deficient mice compared with that in the control mice (Figure 6C and Supplemental Figure 6, F–H). Tumor cell proliferation and tissue hypoxia were attenuated in endothelial SPEN–deficient mice (Figure 6, D and E). To evaluate metastasis, LLC tumors were resected on 14th day after inoculation, when tumors were grossly comparable between control and SPEN-deficient mice, and the mice were maintained for 28 more days. Endothelial SPEN deficiency markedly reduced lung metastasis, consistent with decreased circulating tumor cells (Figure 6, F and G, and Supplemental Figure 6I). Overall survival was extended in e*Spen^–/–^* mice (Figure 6H). These data demonstrate that endothelial SPEN deficiency represses tumor growth and metastasis.

### Endothelial Spen ablation leads to tumor vessel normalization.

**Figure 7:**
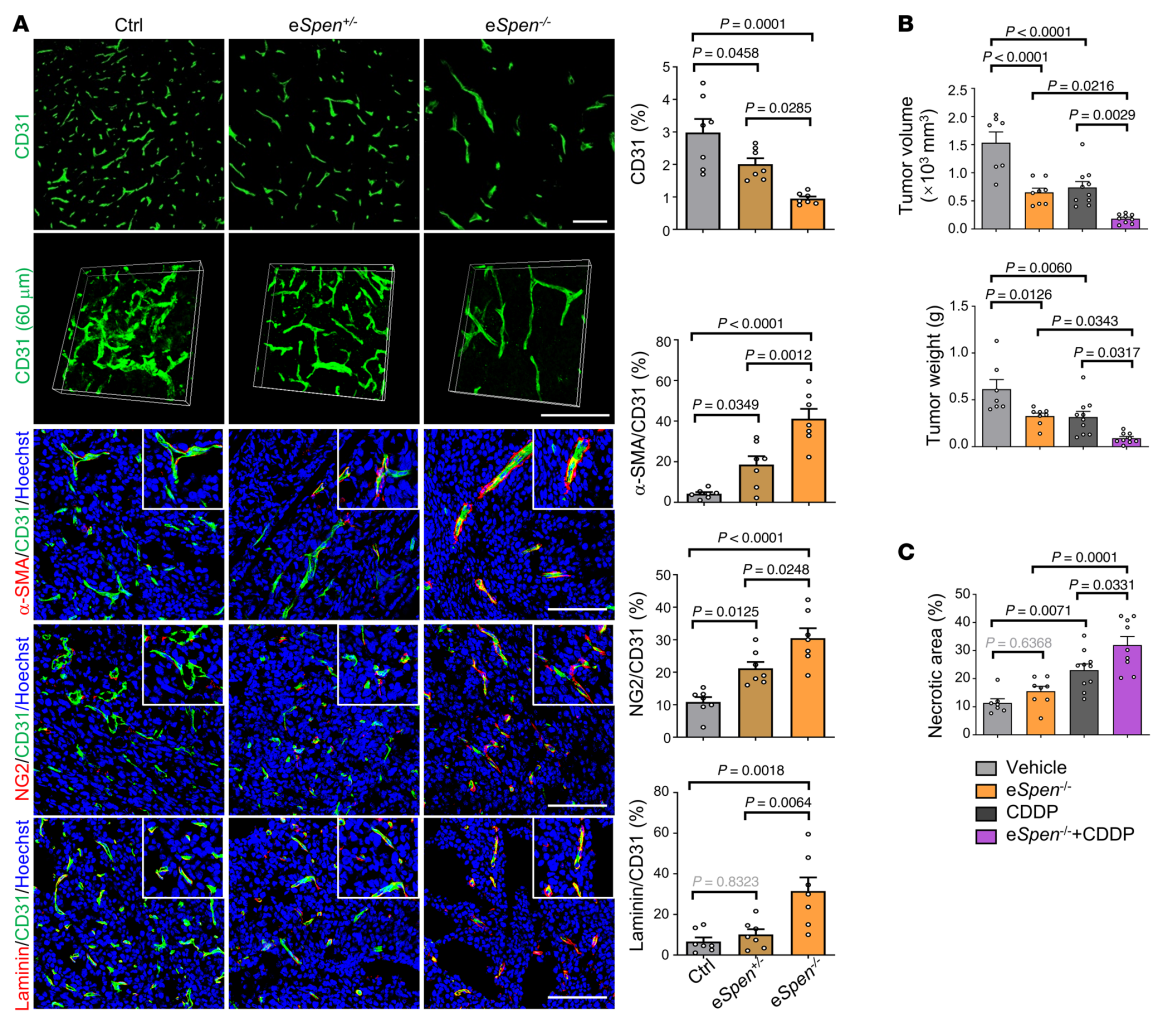
Endothelial *Spen* ablation induces tumor vessel normalization. (**A**) LLC tumors from control, e*Spen^+/–^*, and e*Spen^–/–^* mice were stained for CD31, α-SMA, NG2, and laminin by immunofluorescence on day 21 after inoculation. CD31^+^, α-SMA^+^CD31^+^, NG2^+^CD31^+^, and laminin^+^CD31^+^ areas were quantitatively compared (*n* = 7). The CD31 immunofluorescence (60 μm thickness) was used to reconstruct tumor vessels (representing 3 independent experiments). Scale bars: 100 μm. (**B** and **C**) Mice bearing LLC tumors were treated with CDDP from day 7 after inoculation. (**B**) Tumor size and weight were evaluated on day 21 after inoculation, and (**C**) tumor sections were stained with H&E (Supplemental Figure 7D), and necrosis areas were determined (*n* = 7, 8, 10, and 9 for control, e*Spen^–/–^*, CDDP, and e*Spen^–/–^*+CDDP, respectively). Data represent mean ± SEM. One-way ANOVA with Tukey’s multiple comparisons test was used.

We evaluated tumor vessel phenotype under endothelial SPEN deficiency. Immunostaining of CD31, α-SMA, NG2, and laminin showed that tumor vessel density decreased, accompanied by more regularly organized vasculature, as shown by vessel reconstruction, and increased pericytes and basement membrane coverage in e*Spen^+/–^* and e*Spen^–/–^* mice, suggesting normalized tumor vessels (Figure 7A). A similar phenotype was observed in e*Spen^–/–^* mice inoculated with B16-F10 cells (Supplemental Figure 7, A–C). Cisplatin (CDDP) is one of the most widely used chemotherapeutics in cancer. Tumor vessel normalization is expected to enhance the efficacy of CDDP in tumor treatment (51). We treated tumor-bearing mice of different genotypes with CDDP. The results showed that endothelial SPEN deficiency markedly enhanced the efficacy of CDDP, as shown by reduced tumor growth and increased tumor tissue necrosis (Figure 7, B and C, and Supplemental Figure 7D).

**Figure 8:**
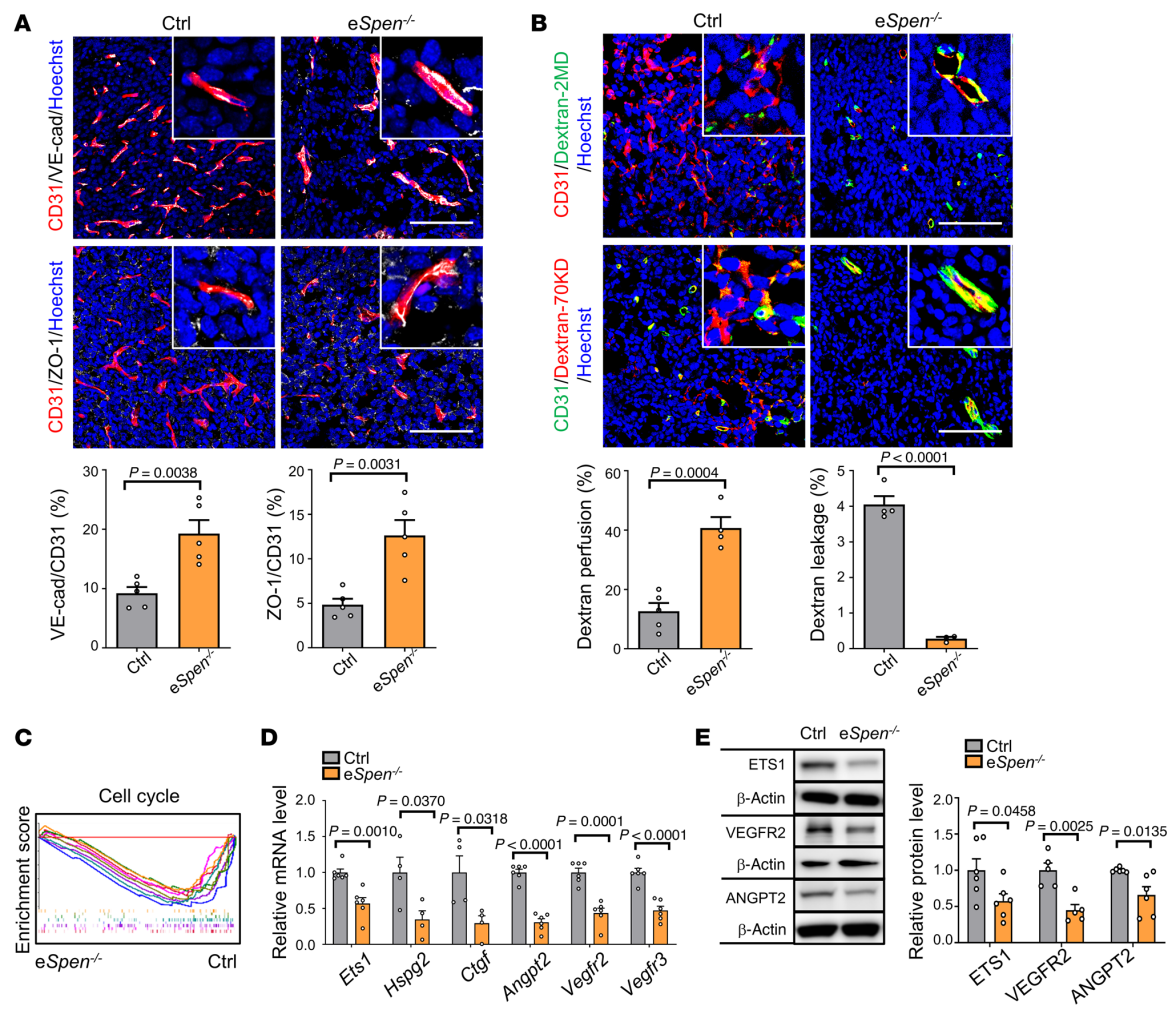
Endothelial *Spen* ablation normalizes functionally tumor vessels. (**A**) LLC tumor sections from control and e*Spen^–/–^* mice were stained with CD31 and VE-cadherin or CD31 and ZO-1 immunofluorescence and quantitatively compared (*n* = 5). Scale bars: 100 μm. (**B**) Vessel perfusion and leakage in tumors were determined with FITC dextran 2 MD (*n* = 5 and 4 for control and e*Spen^–/–^*, respectively) or Texas Red–dextran 70 KD (*n* = 4 and 3 for control and e*Spen^–/–^*, respectively). Scale bars: 100 μm. (**C**) Control and e*Spen^–/–^* TECs were subjected to RNA-Seq. Cell cycle–associated gene sets were analyzed by GSEA (color-coded gene sets are listed in Supplemental Figure 1H). (**D** and **E**) Expression of angiogenesis-related genes in TECs was determined by (**D**) RT-qPCR (*n* = 6, except for *n* = 4 in *Hspg2* and *Ctgf*) or (**E**) immunoblotting (*n* = 6 except for *n* = 5 for VEGFR2). β-Actin served as the loading control. Data represent mean ± SEM. Unpaired 2-tailed Student’s *t* test was used.

At the molecular level, SPEN deficiency increased the expression of EC junctional proteins VE-cadherin and ZO-1 (Figure 8A). Functionally, endothelial *Spen* ablation increased vessel perfusion and reduced leakage (Figure 8B). Consistent with in vitro data, RNA-Seq showed that SPEN-deficient TECs exhibited reduced expression of cell cycle–related genes and angiogenesis-related genes, as confirmed by RT-qPCR and immunoblotting (Figure 8, C–E, and Supplemental Figure 7, E and F). These results indicate that endothelial SPEN deficiency results in tumor vessel normalization.

### p53 deficiency abrogates Spen ablation–induced tumor vessel normalization.

**Figure 9:**
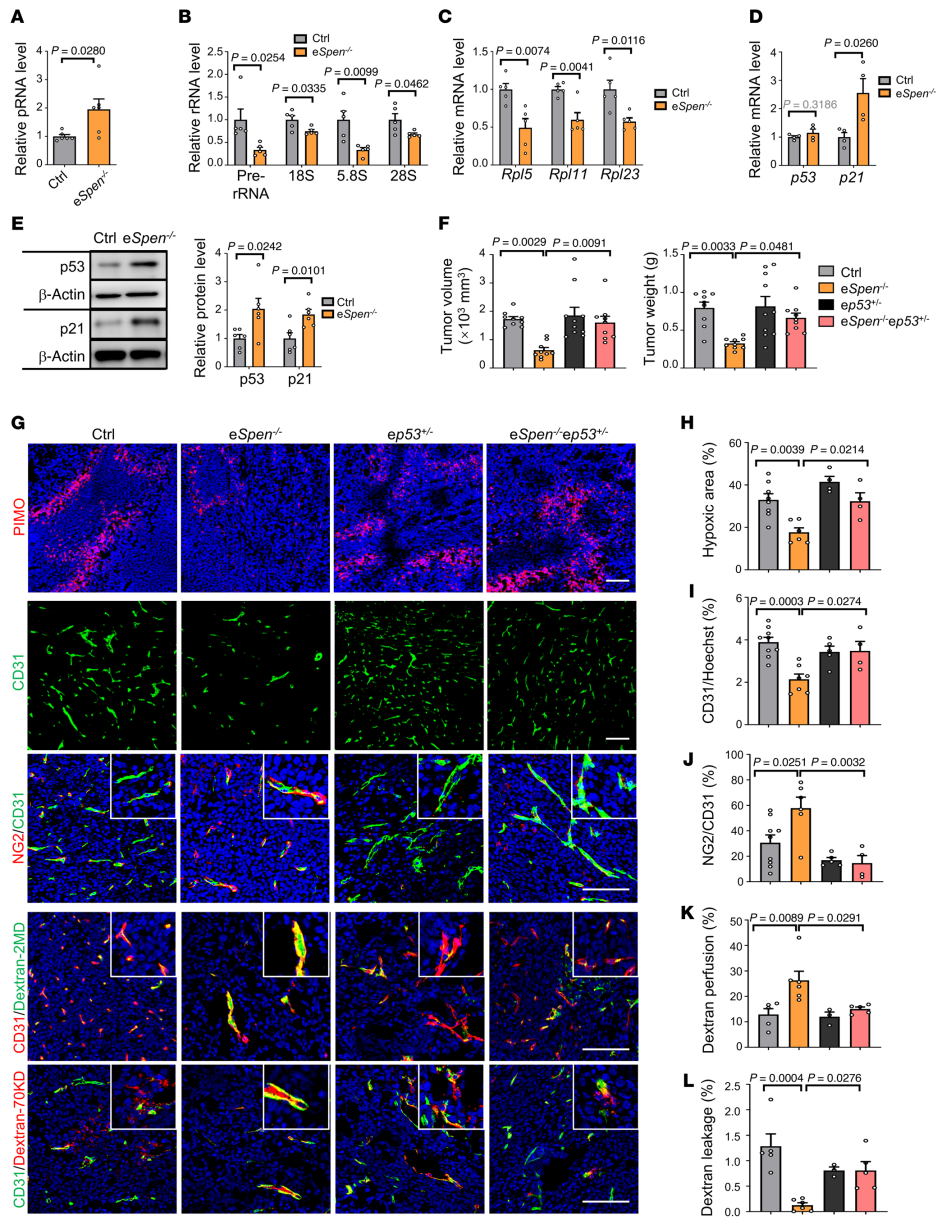
*Spen* ablation–mediated tumor vessel normalization requires p53. (**A**) pRNA expression in TECs, as determined by strand-specific RT-qPCR (*n* = 6). (**B** and **C**) Expression of (**B**) pre-rRNA, 18S, 5.8S, and 28S rRNAs as well as (**C**) *Rpl5*, *Rpl11*, and *Rpl23* in TECs was determined by RT-qPCR (*n* = 5). (**D** and **E**) Expression of *p53* and *p21* in TECs was determined by (**D**) RT-qPCR (*n* = 4) and (**E**) immunoblotting (*n* = 6). β-Actin served as the loading control. (**F**) Mice with different genotypes were inoculated with LLC cells. Tumors were dissected on day 21 after inoculation (Supplemental Figure 8E). Tumor size and weight were quantified (*n* = 9, 9, 10, and 9 for control, e*Spen^–/–^*, e*p53^+/–^*, and e*Spen^–/–^*e*p53^+/–^*, respectively). (**G**) LLC tumors on day 21 after inoculation were stained with Hypoxyprobe, immunofluorescence, or assayed for vessel perfusion and leakage with FITC dextran 2MD or Texas Red–dextran 70 KD. Scale bar: 100 μm. (**H**) The hypoxia (*n* = 8, 6, 4, and 4 for control, e*Spen^–/–^*, e*p53^+/–^*, and e*Spen^–/–^*e*p53^+/–^*, respectively), (**I**) vessel density (CD31^+^) (*n* = 9, 7, 5, and 4 for control, e*Spen^–/–^*, e*p53^+/–^*, and e*Spen^–/–^*e*p53^+/–^*, respectively), (**J**) pericyte coverage (CD31^+^NG2^+^) (*n* = 9, 6, 5, and 4 for control, e*Spen^–/–^*, e*p53*^+/–^, and e*Spen^–/–^*e*p53^+/–^*, respectively) as well as (**K**) vessel perfusion and (**L**) leakage (*n* = 5, 6, 3, and 5 for control, e*Spen^–/–^*, e*p53^+/–^*, and e*Spen^–/–^*e*p53^+/–^*, respectively) were quantified. Data represent mean ± SEM. Unpaired 2-tailed Student’s *t* test was used for **A**–**E** and 1-way ANOVA with Tukey’s multiple comparisons test was used for **F** and **H**–**L**.

In human lung cancer biopsies, in situ hybridization of pRNA and pre-rRNA and SPEN immunofluorescence in TECs showed that a high SPEN level negatively correlated with pRNA level and positively correlated with pre-rRNA level (Supplemental Figure 7, G–I). Then, we examined the expression of rRNA- and p53-related genes in TECs derived from the e*Spen^–/–^* and control mice. KEGG analysis of differentially co-upregulated genes in transcriptomic data of SPEN-deficient TECs and SPENi-transfected HUVECs revealed that the p53 signaling pathway was enriched in the top 20 markedly changed entries, consistent with that p53 signaling is critical for *Spen* ablation–induced tumor vessel normalization (Supplemental Figure 8, A–C). RT-qPCR confirmed that, consistent with in vitro results, pRNA was upregulated, while pre-rRNA, 18S, 5.8S, and 28S rRNAs as well as *Rpl5*, *11*, and *23* mRNAs were concomitantly downregulated in SPEN-deficient TECs (Figure 9, A–C). p53 was upregulated at the protein but not mRNA level, while p21 was upregulated at both the mRNA and protein level in SPEN-deficient TECs (Figure 9, D and E). These results are in line with the idea that endothelial SPEN deficiency represses tumor angiogenesis by activating p53 via nucleolar stress induced by unleashed pRNA expression.

To solidify the role of p53 in *Spen* ablation–induced tumor vessel normalization, we crossed Cdh5-Cre^ERT2^-SPEN^f^ mice with *p53*-floxed (*p53^fl^*) mice to obtain Cdh5-Cre^ERT2^-SPEN^fl/fl^ mice on an endothelial *p53^+/fl^* (e*p53^+/–^*) background. Tamoxifen-induced wild-type (control), e*Spen^–/–^*, e*p53^+/–^*, and e*Spen^–/–^*e*p53^+/–^* mice were inoculated with LLC cells. Heterozygous endothelial p53 disruption (e*p53^+/–^*) almost completely abrogated SPEN disruption–induced (e*Spen^–/–^*-induced) tumor repression (Figure 9F and Supplemental Figure 8, D and E). Immunostaining showed that, while *Spen* ablation resulted in decreased hypoxia accompanied by decreased vessel density and increased pericyte coverage, these phenotypes were reversed by p53 haploinsufficiency (Figure 9, G–J). The p53 haploinsufficiency also canceled *Spen* ablation–induced improvement of vessel function, as determined by the vessel perfusion and leakage assays (Figure 9, G, K, and L). These results demonstrate that SPEN deficiency normalizes tumor vessels by activating p53.

SPEN is a repressor of Notch signaling, which plays a pivotal role in vessel development (22, 52). However, Notch downstream genes hairy/enhancer-of-split related with YRPW motif protein 1 (*HEY1*) and hairy and enhancer of split 1 (*HES1*) were not upregulated in HUVECs or TECs with SPEN knockdown or ablation, respectively (Supplemental Figure 8, F and G), suggesting that SPEN does not repress but rather is required for the canonical Notch signaling in ECs. Double knockout of *Spen* and recombination signal binding protein for immunoglobulin κ J region (*Rbpj*) (53), the transcription factor mediating Notch signaling, did not rescue the *Spen* ablation phenotype (Supplemental Figure 8, H and I), suggesting that SPEN deficiency does not normalize tumor vessels by activating Notch.

### The RNPI inhibitor CX-5461 normalizes tumor vessels and improves chemotherapy.

**Figure 10:**
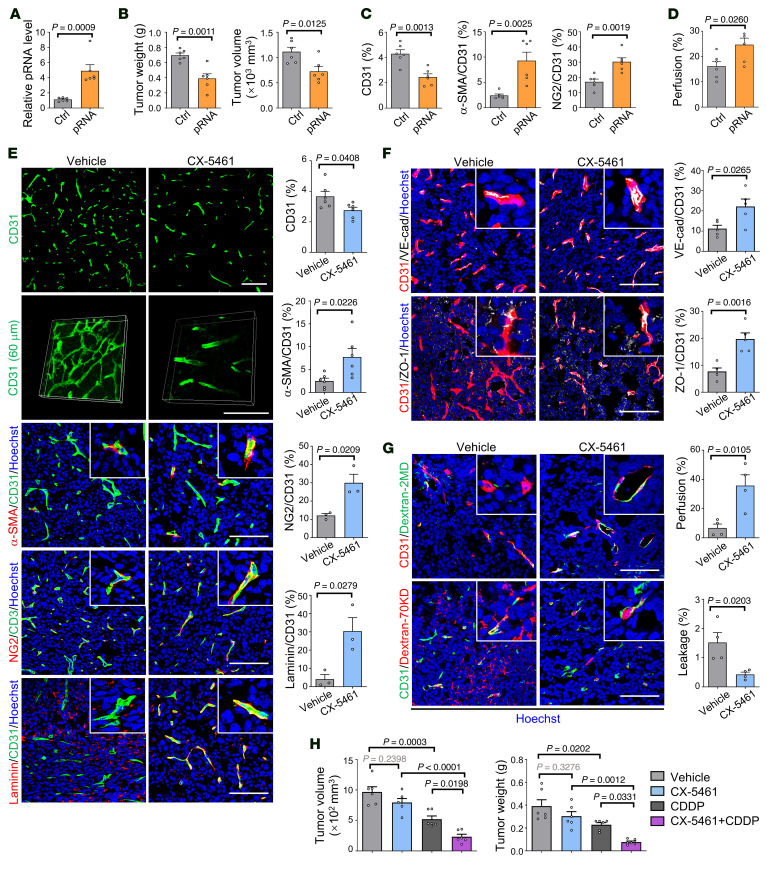
An RNPI inhibitor induces tumor vessel normalization and enhances efficacy of cisplatin. (**A**–**D**) Mice were inoculated with LLC cells and injected with LNP-pRNA or LNP-control (LNP-Ctrl) i.v. every 3 days from day 7 to day 21 after inoculation. (**A**) pRNA expression in TECs was determined by RT-qPCR. (**B**) Tumor growth was determined. (**C** and **D**) Tumor vessels were stained with immunofluorescence, and vessel perfusion was evaluated (Supplemental Figure 9, F–H) (*n* = 6). (**E**) Mice were inoculated with LLC cells and orally administered 50 mg/kg CX-5461 every 2 days from day 7 to day 14 after inoculation. Tumor sections were immunostained on day 14 after inoculation with anti-CD31 (*n* = 6), anti-CD31 plus anti–α-SMA (*n* = 6), anti-CD31 plus anti-NG2 (*n* = 3), and anti-CD31 plus anti-laminin (*n* = 3). Tumor vessels were reconstructed with CD31 immunofluorescence (60 μm thickness) (representing 3 independent experiments). Scale bars: 100 μm. (**F**) LLC tumor sections from mice treated with CX-5461 were stained with CD31 and VE-cadherin or CD31 and ZO-1 immunofluorescence and quantitatively compared (*n* = 5). Scale bars: 100 μm. (**G**) Vessel perfusion and leakage were assessed (*n* = 4). Scale bars: 100 μm. (**H**) Mice bearing LLC tumors were orally administered 50 mg/kg CX-5461 every 2 days and injected i.p. with CDDP every 3 days from day 7 to day 14 after inoculation. Tumors were dissected (Supplemental Figure 10D), and tumor sizes and weights were compared (*n* = 6). Data represent mean ± SEM. One-way ANOVA with Tukey’s multiple comparisons test was used for **H**, and unpaired 2-tailed Student’s *t* test was used for **A**–**G**.

Our data have suggested that induction of nucleolar stress by SPEN deficiency–induced pRNA upregulation could normalize tumor vessels and therefore serves as a target for AAT. To solidify this finding, we synthesized pRNA ASOs and verified the effect on ECs in vitro (Supplemental Figure 9A). We set up LLC tumors in e*Spen^–/–^* and control mice and injected pRNA ASO intratumorally from day 10 after inoculation. The results showed that, while pRNA ASO slightly promoted tumor growth in the control, it abrogated endothelial SPEN deficiency–induced tumor suppression (Supplemental Figure 9B). Moreover, pRNA ASO partially but substantially reversed SPEN deficiency–induced tumor vessel normalization, as shown by increased vessel density and decreased pericyte coverage (Supplemental Figure 9C). Furthermore, we constructed liposome nanoparticles (LNPs) conjugated with cyclo (Arg-Gly-Asp-D-Tyr-Lys) peptide (c(RGDyK)), which targets αvβ3 integrin receptors with high affinity on TECs (54, 55). The LNP was loaded with a plasmid expressing pRNA (LNP-pRNA), which could be taken by TECs and increased pRNA level in TECs after infusion (Figure 10A and Supplemental Figure 9, D and E). Infusion of LNP-pRNA or LNP-control into tumor-bearing mice showed that LNP-pRNA mildly repressed tumor growth and tumor vessel density decreased, while pericyte coverage and vessel perfusion were improved in LNP-pRNA-treated mice (Figure 10, B–D, and Supplemental Figure 9, F–H). These results suggest that upregulating pRNA could normalize tumor vessels while downregulating pRNA has the opposite effect.

Because rRNA gene transcription is driven by RNPI, we assessed whether CX-5461, an RNPI inhibitor under clinical trial, could induce tumor vessel normalization (27–30). Treating HUVECs with CX-5461 downregulated pre-rRNA and upregulated *p21* dose-dependently, with an enlarged cell size resembling that observed under SPEN knockdown (Supplemental Figure 10, A and B). CX-5461 suppresses tumor growth in mice (27). To exclude the proliferation inhibition of CX-5461 on tumor cells, which may influence tumor vessels, we tried different dosing schedules and found that when mice bearing LLC tumors were orally administered with 50 mg/kg CX-5461 every 2 days from day 7 to day 14 after inoculation, tumor growth did not change significantly (Supplemental Figure 10C). Upon this dosing schedule, tumor tissues showed a normalized vasculature, as manifested by a reduced vessel density, increased pericyte and basement membrane coverage, and improved tumor vasculature, as shown by vessel reconstruction, and increased expression of EC junctional proteins VE-cadherin and ZO-1 (Figure 10, E and F). Moreover, CX-5461 treatment increased vessel perfusion and attenuated leakage (Figure 10G). When CX-5461 and CDDP were applied in combination, CX-5461 enhanced the efficacy of CDDP (Figure 10H and Supplemental Figure 10D). We monitored spleen T and B lymphocytes, which are expect to undergo significant proliferation and likely require enhanced ribosome biogenesis, in mice treated with CX-5461 at the dosage generating the size-matched tumors. The results showed that T and B lymphocytes in spleen were not significantly influenced by CX-5461 in our experiments, although spleen size decreased slightly (Supplemental Figure 10, E–H). These results demonstrate that RNPI inhibition with inhibitors such as CX-5461 induces tumor vessel normalization, and improves chemotherapy.

## Discussion

The tumor vasculature has been a therapeutic target of cancer for decades due to its characteristic abnormal structure and hyperactive TECs. AAT normalizes tumor vasculature, leading to attenuated hypoxia and vessel leakage, improved vessel perfusion, and reduced metastasis, which thereby mitigate tumor malignancy (2, 3). However, because tumors by principle employ physiological mechanisms for angiogenesis, discovering efficient targets for AAT has been a long-term challenge (3, 4, 6). In this study, we have revealed for the first time to our knowledge that ribosome biogenesis is an AAT target (Supplemental Figure 10I). Tumor growth stimulates active angiogenesis, which requires the RNPI-mediated transcription of rRNA genes and active ribosome biogenesis in TECs (9–11). Activated TECs upregulate their SPEN to facilitate rRNA gene transcription by repressing pRNA, and SPEN is therefore required for tumor angiogenesis. In the absence of SPEN, pRNA is upregulated and rRNA gene transcription is repressed, thereby disrupting ribosomal biogenesis. This triggers the p53-mediated nucleolar stress response, which results in reduced EC proliferation and tumor vessel normalization. Forced pRNA expression or RNPI inhibitors (CX-5461) can mimic the effect of SPEN deficiency in tumor vessels, leading to tumor vessel normalization, which has been shown previously (27–30, 56). It is noteworthy that SPEN haploinsufficiency results in a similar but less severe phenotype as complete *Spen* ablation, suggesting that the effect of ribosome biogenesis inhibition on tumor angiogenesis is dose dependent. Together, our results demonstrate that ribosome biogenesis is a druggable target for AAT of tumors.

AAT targeting RNPI could have several advantages. First, *p53* is highly mutated in cancer cells but largely intact in tumor microenvironment cells including TECs. Therefore, RNPI-targeted AAT could be expected to be effective irrespective of *p53* mutation. p53 has been shown to limit angiogenesis by interfering with the central regulators of hypoxia that mediate angiogenesis and by inhibiting proangiogenic factor production and increasing endogenous angiogenesis inhibitor production (57). Although p53 mediates endothelial senescence and induces endothelial dysfunction under different conditions, its activation has been shown to exert an antiangiogenic effect on tumors (58–61). Second, nucleolar stress induced by SPEN knockdown does not increase the apoptosis of ECs. This is in contrast to AATs disrupting VEGF signaling, which is required for EC survival (1). Increased TEC death can lead to aggravated hypoxia and tumor metastasis (62). The mechanism of EC survival under nucleolar stress could be related with increased autophagy, but further investigations are needed to address this question (63). In addition, we noticed that cell size increases under SPEN deficiency or RNPI inhibition, which could be related with disturbed ribosome biogenesis (64). Finally, our data showed that the combination of CX-5461 and CDDP markedly enhanced the efficacy of CDDP in mice, supporting the use of RNPI inhibitors in combination with other strategies as a treatment for solid tumors. However, considering potential off-target effects of RNPI inhibitors and the complex mechanisms controlling ribosome biogenesis (65), detailed studies are required to define the dosage and time window required for RNPI inhibitors to serve as an efficient adjuvant of other antitumor therapies such as chemotherapy and immunotherapy.

Nucleoli are specialized, membrane-lacking nuclear structures formed by phase separation (66). The major functions of nucleoli include transcribing and processing rRNA and assembling ribosomes (14, 15). To fulfill these tasks, nucleoli are organized into layered structures, and each structural layer accommodates specific biochemical reactions (38, 66). Numerous extrinsic and intrinsic insults disrupt the function and elegant structure of nucleoli, leading to p53-mediated nucleolar stress (16–18). Our data demonstrated that SPEN deficiency resulted in nucleolar stress in ECs, as manifested by the disordered nucleolar structure, reduced RP expression, and p53 activation, which was responsible for endothelial growth arrest and tumor vessel normalization in this study. SPEN possesses several RNA recognition domains and functions as an RNA-binding protein (20, 23, 24). Protein structure prediction suggests that SPEN contains large stretches of intrinsic disordered regions. These two properties are shared by many proteins participating in phase separation (38). However, our immunostaining of HUVECs with SPEN antibodies showed that SPEN localized outside nucleoli. This suggests that SPEN regulates nucleolar function, rather than constitutes their structure. Indeed, we demonstrate that SPEN deficiency reduces rRNA transcription by upregulating pRNA, a lncRNA derived from the spacer promoter, and inhibits the activity of the gene promoter of rDNA repeats. pRNA knockdown with an ASO not only rescues pre-rRNA expression but also compromised p53 activation, suggesting that SPEN normally represses pRNA to maintain rRNA gene expression. This could physiologically balance the active and inactive rDNA repeats in the rDNA array, which is a suggested function of pRNA (46). The reduced rRNA synthesis induced by SPEN deficiency disrupts the assembly of newborn ribosomes, leading to the redirection of RP and the activation of p53 via MDM2 (16–18). However, pRNA transcription is dependent on RNPI but not RNPII (46), and the mechanism by which SPEN represses the RNPI-mediated transcription of spacer promoters in rDNA repeats has not been elucidated. Moreover, p53 expression is under the control of numerous mechanisms, and other mechanisms underlying p53 upregulation could be involved and worth further investigation in the future.

SPEN is a large protein containing several functional domains, including N-terminal RNA recognition domains, a C-terminal SPOC domain, and motifs interacting with transcription factors located between the N- and C-terminals (19, 20, 24). SPEN does not possess DNA-binding domains, so that SPEN fulfills its transcription repressor functions by interaction with recruiting molecules such as lncRNAs or DNA-binding proteins. At spacer promoter regions in which pRNA transcription starts, the factors responsible for SPEN recruitment have not been defined. One possibility is CTCF, which binds rDNA repeats near the spacer promoter and transcription termination site. CTCF influences the topological architecture of rDNA by forming the chromatin conformation required for RNPI recruitment and rDNA transcription. The lncRNA SRA binds to and regulates the function of CTCF (25). SRA also binds SPEN (20). It is therefore possible that SPEN binds CTCF via SRA and influences the conformation of rDNA. Moreover, a recent report showed that SPEN binds directly to endogenous retroviral (ERV) RNAs and participates in ERV silencing (26). Some rDNA repeats are silenced by epigenetic mechanisms, while others remain active (14, 15). Whether SPEN participates in silencing rDNA repeats in a manner similar to that of ERV is worthy of further investigation. Moreover, the SPOC domain provides a protein-interacting platform to recruit transcription repressors, such as HDACs, EZH2, NcoR and m6A modification enzymes (24). It will be interesting to examine the roles of these enzymes in SPEN-mediated nucleolar homeostasis.

It has been demonstrated that SPEN is recruited through its interaction with RBPJ, which thereby results in repression of canonical Notch signaling (22). However, SPEN could also be recruited to chromatin to promote heterochromatin formation and modify gene expression networks at the epigenetic level (24, 26). In ECs, our data showed that the Notch downstream genes *HES1* and *HEY1* were not upregulated under SPEN deficiency, suggesting that SPEN does not repress but is rather required for the canonical Notch signaling in ECs. This is consistent with previous findings in *Drosophila* (67, 68). Functionally, although both SPEN deficiency and RBPJ deficiency inhibit tumor growth, SPEN deficiency normalizes while RBPJ deficiency disrupts tumor vasculature, and disruption of both leads to normalized tumor vessels. Therefore, more studies are required to elucidate the relationship between SPEN and Notch in ECs.

## Methods

### Human samples.

Human lung adenocarcinoma tissue microarrays (HLugA180Su07, HLugA180Su08), human gastric cancer tissue microarray (HStmA180Su30), and human breast cancer tissue microarray (HBreD136Su02) were provided by Shanghai Outdo Biotech Co. Ltd. (Supplemental Tables 1–4).

### Animals.

Mice were maintained in a specific pathogen–free facility. *Spen*-floxed, *Cdh5-Cre^ERT2^* transgenic, and *Rbpj*-floxed mice were described previously (31, 53, 69). *p53*-floxed mice were purchased from Shanghai Model Organisms Center Inc. Mice were backcrossed with C57BL/6J mice for more than 6 generations and genotyped by PCR using tail DNA as a template. To induce Cre-mediated recombination, 6- to 8-week-old male or female mice were injected i.p. with 100 μL tamoxifen (20 mg/mL, MilliporeSigma, T5648), while P1 pups were injected s.c. with 2.5 μL tamoxifen (Supplemental Figure 2B).

For mouse tumor models, LLC (5 × 10^6^) or B16-F10 (1 × 10^6^) cells were inoculated s.c. in the right back of trunks 1 day after the last tamoxifen injection and maintained for 21 or 16 days after inoculation, respectively. Tumor size was monitored using a caliper and calculated as π × [*d*^2^ × *D*]/6, where *d* represents short diameter and *D* denotes long diameter. In some experiments, CDDP (2.5 mg/kg, Selleck, S1166) was injected i.p. every 3 days from 7 days after inoculation. CX-5461 (50 mg/kg, Selleck, S2684) was administered by gastric gavage every 2 days from 7 days after inoculation. LNP (25 μg DNA, 200 μL/mouse, see below) was injected i.v. every 3 days from 7 days after inoculation. The 2′-O-(2-Methoxyethyl) phosphorothioate ASO against mouse pRNA (Shanghai Integrated Biotech Solutions Co. Ltd.) was injected intratumorally at a dosage of 5 nmol per mouse every 3 days from 10 days after inoculation. Three ASO were tested (5′-GGACCTCAAAGGAACAACTG, 5′-CGGAGAACTGATAAGACCGA, and 5′-GGTCCAATAGGAACAGATAG), with the first one used for further study. To evaluate metastasis, LLC cells were transduced with luciferase (luciferase-LLC) or GFP (GFP-LLC) lentivirus (GeneChem). Luciferase-LLC tumors were surgically removed on day 14 after inoculation after anesthetization with 1% pentobarbital sodium. On day 28 after tumor resection, mice were injected with D-luciferin (150 mg/kg, Yeasen, 40902ES01) and sacrificed 8 minutes later. Their lungs were removed, photographed, and analyzed with a bioluminescence imaging system (IVIS Lumina II, Perkin-Elmer), followed by histological staining. To detect circulating tumor cells, the GFP-LLC cells were inoculated, and blood was collected on day 21 after inoculation. After erythrolysis with red lysis buffer (Cwbio, CW0613), GFP^+^ cells were counted under a fluorescence microscope (NI-E, Nikon). For survival analysis, LLC tumors were surgically removed on day 21 after inoculation, and survival of mice was plotted by the Kaplan-Meier method.

A Matrigel plug assay was performed by injecting 0.3 mL Matrigel (Corning, 354230) containing 400 ng/mL VEGF (SinoBio, 50159-MNAB) and 250 ng/mL bFGF (SinoBio, 50037-M07E) into the mouse groin. The plugs were recovered on day 7 and fixed in 4% paraformaldehyde (PFA) overnight. Masson’s trichrome staining was conducted using a kit (Servicebio, G1006).

### Histology.

Tissues were fixed in 4% PFA at 4°C overnight and embedded in paraffin routinely. Samples were cut into 4 μm thick sections and then subjected to H&E staining. Fluorescence triple staining was conducted using a TSAPLus Fluorescence Triple Staining Kit (Servicebio, G1236).

For immunofluorescence, tissues were fixed in 4% PFA at 4°C for 4 hours, followed by dehydration in 30% sucrose-PBS overnight. The samples were then embedded in optimal cutting temperature compound (Sakura, 4583). Frozen blocks were sectioned at 10 μm or 60 μm thickness, dried at room temperature for 2 hours, and blocked with PBS containing 5% BSA and 0.3% Triton X-100 for 1 hour at room temperature. The samples were incubated overnight at 4°C with primary antibodies. After washing, the sections were incubated with secondary antibodies at room temperature for 2 hours and counterstained with Hoechst (MilliporeSigma, 94403) for 15 minutes at room temperature. Cell samples on coverslips were fixed with 4% PFA for 30 minutes and blocked with PBS containing 5% BSA and 0.3% Triton X-100 for 30 minutes at room temperature. For whole-mount retinal staining, eyeballs were harvested and fixed in 4% PFA at 4°C for 2 hours, and the retinas were dissected and stained as described above. EdU labeling was performed by injecting i.p. EdU (50 μg/g, RiboBio, C00053) 4 hours before euthanasia and staining using the Cell Light EdU Apollo 567 In Vitro Kit (RiboBio, C10310-1). RNA-ISH combined with fluorescent IHC was conducted using the RNA-Protein Co-Detection Ancillary Kit (323180; ACD Bio) according to the manufacturer’s protocol. The human pre-rRNA and pRNA (+551 to +2,922 and –415 to –32, respectively, Genebank, U13369.1) probes were ordered from ACD Bio. Images were captured under a fluorescence microscope (NI-E, Nikon), confocal microscope (A1R, Nikon), or SIM microscope (N-SIM S, Nikon). The immunofluorescence staining images of human biopsies were quantified by TissueFAXS Q+ 2D/3D panoramic tissue cell imaging quantitative analysis system. The expression of SPEN, pre-rRNA, and pRNA in CD31^+^ ECs was quantified using IMARIS 9.0.1. Antibodies used are listed in Supplemental Table 5.

To detect hypoxia, mice were injected i.p. with pimonidazole hydrochloride (60 mg/kg, Cayman, 89130) 1 hour prior to tumor harvesting. Cryosections were stained with a Hypoxyprobe-1-Mab1 kit (Hypoxyprobe, PAb2627AP). To examine vascular perfusion and leakage, mice were injected i.v. with 5 mg FITC-conjugated dextran 2 MD (MilliporeSigma, FD2000s) or 0.25 mg Texas Red–conjugated dextran 70 KD (Invitrogen, D1864) and perfused by intracardiac infusion with PBS 15 minutes after the injection under anesthetization. Immunostaining was conducted as described above.

For transmission electron microscopy (TEM), cells were trypsinized and fixed first in 2.5% glutaraldehyde and then in ferrocyanide-reduced osmium tetroxide. After uranyl staining *en bloc*, samples were embedded in epoxy resin according to standard procedures. Ultrathin sections were obtained and observed under an electron microscope (Tecnai Spirit of FEI or JEM-1230, Japan Electronics Co. Ltd.).

### Cell culture and transfection.

LLC, B16-F10, and HEK-293T cells were obtained from ATCC and authenticated by both morphological analysis and short tandem repeat profiling. Cells were maintained in DMEM supplemented with 10% FCS. HUVECs were cultured in EC medium (ScienCell, 1001) supplemented with 5% FBS, 1% EC growth supplements, and 1% streptomycin-penicillin. HUVECs were used between passages 2 and 6. CX-5461 was applied at different concentrations, with 50 mM NaH_2_PO_4_ as the vehicle. Cycloheximide was used at the final concentration of 20 μM.

To isolate primary ECs, normal or tumor tissues were minced mechanically and digested in 1 mg/mL collagenase I (MilliporeSigma, C0130) and 100 μg/mL DNase I (Roche, 10104159001) for 30 minutes at 37°C. After passing through a 70 μm tissue strainer, cell suspensions were centrifuged for 4 minutes at 350*g* at 4°C, followed by erythrolysis. The cells were resuspended in 90 μL PBS containing 0.5% BSA and 2 mM EDTA and mixed with 10 μL anti-CD31–coated magnetic beads (Miltenyi, 130-097-418). After incubation at 4°C for 30 minutes, the cells were collected using a magnetic bead collector (Miltenyi) and then washed 3 times with PBS containing 0.5% BSA and 2 mM EDTA. ECs were evaluated by flow cytometry after staining with anti-endomucin.

Transfection of ECs with shRNA for *SPEN* (shRNA1 5′-CCAGTACGCTCTACAGATA and SPENi 5′-CCCGATCACGCCGCAAGCGAA), *p53* (5′-CGGCGCACAGAGGAAGAGAAT), *p21* (5′-AAGACCATGTGGACCTGTCAC), or the NC was achieved by lentiviral transduction at the multiplicity of infection (MOI) of 10. Transduction was performed on day 0, and the culture medium was replaced with fresh medium 24 hours later. Overexpression was achieved by adenovirus transduction at the MOI of 200, and the culture medium was replaced with fresh medium 4 hours later. Lentivirus or adenovirus construction and packaging were conducted by GeneChem and Vigene Biosciences. The ASO of human pRNA (5′-GGACACCTGTCCCCAAAAAC) was transfected with HiPerFect Transfection Reagent (Qiagen, 301705) at a final concentration of 100 nM.

Endogenous *SPEN* gene was activated using the lentivirus CRISPR-Cas9 Synergistic Activation Mediator system (Genechem Co. Ltd.) following the manufacturer’s protocol (70). Briefly, HUVECs were infected with lentiviruses encoding dCas9-VP64 (lenti-dCAS9-VP64-Puro) and sgRNAs (lenti-sgRNA-MS2-P65-HSF1-Neo) simultaneously at the MOI of 5, and the culture medium was replaced with fresh medium 24 hours later. Activation of *SPEN* expression was determined on day 4. Cells infected with dCas9-VP64 and nontargeting sgRNA lentiviruses were used as controls. Three sgRNAs (5′-TAGTCCCTCACTTCGTCGCC, 5′-GCTAGTGGAGTCCCGCTGCT, and 5′-ACGAAGTGAGGGACTACAGG) were tested, and the third one (SPEN^OE3^) was used for further study.

For reporter assay, HEK-293T cells in 48-well plates (5 × 10^3^ cells/well) were transduced with SPENi lentivirus. The cells were then transfected with 200 ng of the p53 reporter plasmid (p53-luc, Yeasen, 11540ES03) and 10 ng pRL-TK (Promega, E2241). The cells were harvested 24 hours after transfection, and the luciferase activity was analyzed with the Dual-Luiferase Reporter Assay System (Promega).

### Time-lapse imaging.

Cells were sparsely seeded in a quartered confocal dish well or 6-well plate. Time-lapse images were recorded using a live-cell imaging workstation under a confocal microscope at 3-minute intervals or a fluorescence microscope at 5-minute intervals. The velocity of movement was determined by Fiji v2.0.0 with the Trackmate plugin.

### Cell proliferation and migration.

HUVECs were cultured in fresh ECM containing 1% FBS for 24 hours and then in ECM with 5% FBS for an additional 24 hours. The cells were then cultured in medium containing 50 μM EdU (RiboBio, C10310-1) for 2 hours, fixed with 4% PFA at room temperature for 30 minutes, and stained with a Cell Light EdU Apollo 567 In Vitro Kit (RiboBio, C10310-1). Images were captured under a fluorescence microscope.

For migration, cells were seeded in 24-well plates at 1 × 10^5^ cells/well and allowed to reach confluence over the next 24 hours. A scratch was made using a pipette tip, and the closure of the scratch was monitored for 12 hours in ECM containing 1% FBS.

### Fibrin bead sprouting assay.

HUVECs expressing EGFP were cultured in fresh EGM-2 medium (Lonza, CC-3162). HUVECs were incubated with Cytodex 3 microbeads (400 cells per bead, MilliporeSigma, C3275) at 37°C for 4 hours and then transferred into 12-well plates containing EGM-2 medium and cultured overnight. The next day, microbeads were embedded in fibrinogen (MilliporeSigma, F4883) containing 0.625 U/mL thrombin (MilliporeSigma, T4648) and 0.15 U/mL aprotinin (MilliporeSigma, A1153) at a density of 100 beads/mL in a 48-well plate, and 0.5 mL EGM-2 medium was added to mouse lung fibroblasts (MRC5) (1 × 10^4^ cells/well). The cells were cultured for 4 days with two medium changes. Images were captured under a fluorescence microscope, and sprouting was quantified by counting the number or length of sprouts.

### RT-qPCR.

Total RNA was extracted using TRIzol reagent (Invitrogen, 15596018). cDNA was synthesized with a reverse transcription kit (Takara, RR036A). Real-time PCR was conducted using a SYBR Premix Ex Taq Kit (Takara, RR820A) on an ABI QuantStudio 5 real-time PCR system (Thermo Fisher Scientific), with β-actin as an internal control. For strand-specific RT-qPCR, RNA was extracted with the RNAprep Pure Kit (Tiangen, DP430), and genomic DNA was removed with RNase-free DNase I. Strand-specific primers were used to synthesize sense or antisense chains using the Transcriptor First Strand cDNA Synthesis Kit (Roche, 4897030001), followed by real-time PCR. The 7SK sense transcript was used as a control. Primers used are listed in Supplemental Table 6.

### ChIP-qPCR assay.

ChIP was performed using a SimpleChIP Enzymatic Chromatin IP Kit (Cell Signaling Technology, 9003). Briefly, HUVECs were treated with 1% formaldehyde. Crosslinked chromatin was digested with micrococcal nuclease for 20 minutes at 37°C and sonicated. Antibodies or control IgG was applied to pull down fragmented chromatin, and chromatin-antibody complexes were collected with protein-G beads and washed extensively. After elution, DNA-protein crosslinks were reversed by incubation at 65°C for 2 hours. Precipitated DNA fragments were extracted and analyzed by qPCR, and the results were normalized to those of the genomic DNA preparations. Primers used are listed in Supplemental Table 6.

### FACS.

Cells were collected routinely. After erythrolysis, cells were resuspended in PBS containing 2% inactivated FBS and 0.1% NaN_3_ and stained in the dark for 30 minutes with antibody cocktails on ice. Analysis was performed on a FACS Canto II instrument (BD Pharmingen). Cell viability was evaluated with 7-amino-actinomycin D (BD Pharmingen, 559925). Data were analyzed using FlowJo V.10 software (TreeStar). Antibodies used are listed in Supplemental Table 5.

For cell cycle analysis, HUVECs were trypsinized and fixed in 70% ethanol overnight. The fixed cells were incubated in PBS containing 0.2% Triton X-100, 100 μg/mL RNase A (Roche, 10109142001), and 50 μg/mL propidium iodide for 30 minutes at 37°C and analyzed with a FACSCalibur flow cytometer (BD Biosciences) or CytoFLEX flow cytometer (Beckman Coulter).

### Preparation of cationic lipid nanoparticles.

To prepare lipid nanoparticles (Xi’an Ruixi Biological Technology Co. Ltd.) (54, 55), 60 mg soybean lecithin, 6 mg N-[1-(2,3-dioleoyloxy)-propyl]-N,N,N-trimethylammonium methyl-sulfate, 1.2 mg 1.2-distearoyl-sn-glycero-3-phosphoethanolamine-N-[methoxy (polyethylene glycol)-2000] (DSPE-PEG2000), 2.4 mg 1,2-distearoyl-sn-glycero-3-phosphoethanolamine-N-[methoxy(polyethylene glycol)-2000]-c(RGDyK) (DSPE-PEG2000-cRGD), and 3.6 mg cholesterol were dissolved in 2 mL ethanol and transferred to solanum-shaped flask. Plasmids (pcDNA3.1, pcDNA3.1-pRNA [–232 to –1 of mouse rDNA, Genebank BK000964.3], refs. 71, 72, or pIRES2-dsRED) DNA (3 mg) were dissolved in 50 mM citrate buffer (pH4.0) containing 25% ethanol, and then slowly added into the flask. After 20 minutes of incubation, the mixture was treated with ultrasound and liposome extruder (100 nm filter). Free DNA was removed by a nanodialysis device. The encapsulation efficiency was between 86% and 91%.

### RNA-Seq.

Total RNA was extracted using TRIzol reagent from HUVECs or primary TECs. RNA quality was evaluated using an Agilent 2200 Tape Station and RNase-free agarose gel electrophoresis. mRNA was enriched with oligo(dT) beads, fragmented with fragmentation buffer, and reverse transcribed with random primers. Second-strand cDNA was synthesized, and the cDNA fragments were purified with a QIAquick PCR extraction kit (Qiagen), end repaired, and ligated to Illumina sequencing adapters. The ligation products were size-selected by agarose gel electrophoresis, amplified, and sequenced on an Illumina NovaSeq6000 platform for HUVECs (Gene Denovo Biotechnology Corporation) and on an Illumina Xten platform for TECs (Annoroad). Principal component analysis was performed based on the fast.prcomp function of gmodels in R package (version 3.6.0), where the parameter is set to scale = f and center = t. After dimensionality reduction, the PCs were ranked based on the percentage of variance by each PC, and the first two PCs were extracted to draw a scatter plot with the geom_point function in ggplot2 package (version 2_3.3.5). Other bioinformatic analyses, including the differential gene expression analysis, pathway analysis, and gene set enrichment analysis (GSEA), were performed using the OmicShare tools (73), a free online platform for data analysis, TB Tools software and GSEA2.2.4.

### Immunoblotting.

Cell lysates were prepared with RIPA buffer (Beyotime, P0013B) containing 1 mM phenylmethanesulfonyl fluoride (Thermo Fisher Scientific, 36978). Proteins were separated by SDS-PAGE and blotted onto polyvinylidene fluoride membranes. The membranes were blocked with 5% skim milk in PBS-0.1% Tween 20 and then incubated with primary antibodies at 4°C overnight, followed by washing and incubation with secondary antibodies at room temperature for 2 hours. After washing, the blots were developed with enhanced chemiluminescence (Thermo Fisher Scientific) and detected using a ChemiScope Imaging System (Clinx Science Instruments). β-Actin was used as an internal reference. A Nuclear and Cytoplasmic Protein Extraction Kit (Beyotime, P0028) was used to separate nuclear and cytoplasmic proteins, with β-actin and lamin A/C serving as references for cytoplasmic and nuclear proteins, respectively.

### Immunoprecipitation.

Cell lysates were prepared and quantified. Primary antibodies (10 μg) or isotype control were incubated with 30 μL Protein-G magnetic beads (Invitrogen, 10004D) for 2 hours at 4°C with gentle rotation. Then, the antibody-coated beads were mixed with cell lysates with equal amounts of total proteins and incubated at 4°C overnight. The beads were washed 3 times with ice-cold PBS and boiled for 15 minutes in reductive loading buffer before SDS-PAGE and immunoblotting. For the detection of the MDM2/RPL5/RPL11/5S rRNA complex, cell lysates were first subjected to ultracentrifugation at 20,0000*g* for 2 hours at 4°C, followed by routine immunoprecipitation and detection (74).

### Statistics.

Quantitative analysis was performed using Image-Pro Plus 6.0, Fiji v2.0.0, IMARIS 9.0.1, FlowJo 7.6.1, and FlowJo V.10 software. Statistical analysis was performed using GraphPad Prism 8.0 software. All quantitative data are presented as the mean ± SEM. Statistical significance was calculated using the unpaired 2-tailed *t* test (for 2 groups) or 1-way ANOVA with Tukey’s multiple comparison test (for more than 2 groups). χ^2^ Analyses were performed to compare the distributions of tumor stages among the human lung adenocarcinoma tissue cohort. Log-rank (Mantel-Cox) tests were used for survival analysis. Correlation between SPEN and RNA expression in the human lung adenocarcinoma tissue cohort was determined by Spearman’s rank-order correlation analysis. *P* values of less than 0.05 were considered statistically significant.

### Study approval.

Protocols involving human samples were approved by the ethics committee of Xijing Hospital, Fourth Military Medical University. The animal experiments were approved by the Animal Experiment Administration Committee of Fourth Military Medical University.

### Data availability.

Original RNA-Seq data are available in the Genome Sequence Archive (GSA) database (https://bigd.big.ac.cn/gsa), with accessions HRA000788 for HUVECs and CRA004085 for TECs. Values for all data points in graphs are reported in the Supporting Data Values file.

## Author contributions

ZYY, XCY, and JYLZ performed experiments and collected data. LL assisted with experiments and data collection. CCG, PRZ, BR, and JLD helped with cell and molecular biology experiments. YL, JXS, RNW, XXF, BC, and TX assisted with animal maintenance and experiments. HH designed the experiments and prepared the manuscript. ZYY, XCY, and JYLZ share first authorship, and the order in which they are listed was determined by workload.

## Supplementary Material

Supplemental data

Supplemental video 1

Supplemental video 2

Supporting data values

## Version 1 08/22/2023

In-Press Preview

## Version 2 10/16/2023

Electronic publication

## References

[1] Carmeliet P, Jain RK (2011). Molecular mechanisms and clinical applications of angiogenesis. Nature.

[2] Lugano R (2020). Tumor angiogenesis: causes, consequences, challenges and opportunities. Cell Mol Life Sci.

[3] Martin JD (2019). Normalizing function of tumor vessels: progress, opportunities, and challenges. Annu Rev Physiol.

[4] Jászai J, Schmidt MHH (2019). Trends and challenges in tumor anti-angiogenic therapies. Cells.

[5] Cao Y (2011). Forty-year journey of angiogenesis translational research. Sci Transl Med.

[6] Li X (2019). Hallmarks of endothelial cell metabolism in health and disease. Cell Metab.

[7] Itatani Y (2018). Resistance to anti-angiogenic therapy in cancer-alterations to anti-VEGF pathway. Int J Mol Sci.

[8] Haibe Y (2020). Resistance mechanisms to anti-angiogenic therapies in cancer. Front Oncol.

[9] Goveia J (2020). An integrated gene expression landscape profiling approach to identify lung tumor endothelial cell heterogeneity and angiogenic candidates. Cancer Cell.

[10] Zhao Q (2020). Heterogeneity and chimerism of endothelial cells revealed by single-cell transcriptome in orthotopic liver tumors. Angiogenesis.

[11] Rohlenova K (2020). Single-cell RNA sequencing maps endothelial metabolic plasticity in pathological angiogenesis. Cell Metab.

[12] Pelletier J (2018). Ribosome biogenesis in cancer: new players and therapeutic avenues. Nat Rev Cancer.

[13] Piazzi M (2019). Signal transduction in ribosome biogenesis: a recipe to avoid disaster. Int J Mol Sci.

[14] Potapova TA, Gerton JL (2019). Ribosomal DNA and the nucleolus in the context of genome organization. Chromosome Res.

[15] Moss T (2019). The chromatin landscape of the ribosomal RNA genes in mouse and human. Chromosome Res.

[16] Zhang Y, Lu H (2009). Signaling to p53: ribosomal proteins find their way. Cancer Cell.

[17] Yang K (2018). Nucleolar stress: hallmarks, sensing mechanism and diseases. Cell Stress.

[18] Nicolas E (2016). Involvement of human ribosomal proteins in nucleolar structure and p53-dependent nucleolar stress. Nat Commun.

[19] Ariyoshi M, Schwabe JW (2003). A conserved structural motif reveals the essential transcriptional repression function of Spen proteins and their role in developmental signaling. Genes Dev.

[20] Arieti F (2014). The crystal structure of the Split End protein SHARP adds a new layer of complexity to proteins containing RNA recognition motifs. Nucleic Acids Res.

[21] Shi Y (2001). Sharp, an inducible cofactor that integrates nuclear receptor repression and activation. Genes Dev.

[22] Kuroda K (2003). Regulation of marginal zone B cell development by MINT, a suppressor of Notch/RBP-J signaling pathway. Immunity.

[23] Boeren J, Gribnau J (2021). Xist-mediated chromatin changes that establish silencing of an entire X chromosome in mammals. Curr Opin Cell Biol.

[24] Dossin F (2020). SPEN integrates transcriptional and epigenetic control of X-inactivation. Nature.

[25] Yao H (2010). Mediation of CTCF transcriptional insulation by DEAD-box RNA-binding protein p68 and steroid receptor RNA activator SRA. Genes Dev.

[26] Carter AC (2020). Spen links RNA-mediated endogenous retrovirus silencing and X chromosome inactivation. Elife.

[27] Drygin D (2011). Targeting RNA polymerase I with an oral small molecule CX-5461 inhibits ribosomal RNA synthesis and solid tumor growth. Cancer Res.

[28] Bywater MJ (2012). Inhibition of RNA polymerase I as a therapeutic strategy to promote cancer-specific activation of p53. Cancer Cell.

[29] Khot A (2019). First-in-human RNA polymerase I transcription inhibitor CX-5461 in patients with advanced hematologic cancers: results of a phase I dose-escalation study. Cancer Discov.

[30] Sanij E (2020). CX-5461 activates the DNA damage response and demonstrates therapeutic efficacy in high-grade serous ovarian cancer. Nat Commun.

[31] Yabe D (2007). Generation of a conditional knockout allele for mammalian Spen protein Mint/SHARP. Genesis.

[32] Bieging KT (2014). Unravelling mechanisms of p53-mediated tumour suppression. Nat Rev Cancer.

[33] Geyer RK (2000). The MDM2 RING-finger domain is required to promote p53 nuclear export. Nat Cell Biol.

[34] ElSawy KM (2015). A spatiotemporal characterization of the effect of p53 phosphorylation on its interaction with MDM2. Cell Cycle.

[35] Liu Y (2019). p53 modifications: exquisite decorations of the powerful guardian. J Mol Cell Biol.

[36] Mantovani F (2015). Interaction of p53 with prolyl isomerases: Healthy and unhealthy relationships. Biochim Biophys Acta.

[37] Jeong K (2014). p53 negatively regulates Pin1 expression under ER stress. Biochem Biophys Res Commun.

[38] Lafontaine DLJ (2021). The nucleolus as a multiphase liquid condensate. Nat Rev Mol Cell Biol.

[39] Wang X (2021). Mutual dependency between lncRNA LETN and protein NPM1 in controlling the nucleolar structure and functions sustaining cell proliferation. Cell Res.

[40] Zentner GE (2011). Integrative genomic analysis of human ribosomal DNA. Nucleic Acids Res.

[41] Herdman C (2017). A unique enhancer boundary complex on the mouse ribosomal RNA genes persists after loss of Rrn3 or UBF and the inactivation of RNA polymerase I transcription. PLoS Genet.

[42] Mars JC (2018). A deconvolution protocol for ChIP-seq reveals analogous enhancer structures on the mouse and human ribosomal RNA genes. G3 (Bethesda).

[43] Hamdane N (2014). Conditional inactivation of upstream binding factor reveals its epigenetic functions and the existence of a somatic nucleolar precursor body. PLoS Genet.

[44] Huang K (2013). Ribosomal RNA gene transcription mediated by the master genome regulator protein CCCTC-binding factor (CTCF) is negatively regulated by the condensin complex. J Biol Chem.

[45] Van de Nobelen S (2010). CTCF regulates the local epigenetic state of ribosomal DNA repeats. Epigenetics Chromatin.

[46] Mayer C (2006). Intergenic transcripts regulate the epigenetic state of rRNA genes. Mol Cell.

[47] Bierhoff H (2010). Noncoding transcripts in sense and antisense orientation regulate the epigenetic state of ribosomal RNA genes. Cold Spring Harb Symp Quant Biol.

[48] Zhao Z (2018). lncRNA PAPAS tethered to the rDNA enhancer recruits hypophosphorylated CHD4/NuRD to repress rRNA synthesis at elevated temperatures. Genes Dev.

[49] Abraham KJ (2020). Nucleolar RNA polymerase II drives ribosome biogenesis. Nature.

[50] Vydzhak O (2020). Non-coding RNAs at the eukaryotic rDNA locus: RNA-DNA hybrids and beyond. J Mol Biol.

[51] Leite de Oliveira R (2012). Gene-targeting of Phd2 improves tumor response to chemotherapy and prevents side-toxicity. Cancer Cell.

[52] Zhang N (2021). DLL1 orchestrates CD8^+^ T cells to induce long-term vascular normalization and tumor regression. Proc Natl Acad Sci U S A.

[53] Han H (2002). Inducible gene knockout of transcription factor recombination signal binding protein-J reveals its essential role in T versus B lineage decision. Int Immunol.

[54] Reschke M (2022). Nucleic acid delivery to the vascular endothelium. Mol Pharm.

[55] Sakurai Y (2014). RNAi-mediated gene knockdown and anti-angiogenic therapy of RCCs using a cyclic RGD-modified liposomal-siRNA system. J Control Release.

[56] Ye Q (2017). Therapeutic targeting of RNA polymerase I with the small-molecule CX-5461 for prevention of arterial injury-induced neointimal hyperplasia. Arterioscler Thromb Vasc Biol.

[57] Men H (2021). The regulatory roles of p53 in cardiovascular health and disease. Cell Mol Life Sci.

[58] Warboys CM (2014). Disturbed flow promotes endothelial senescence via a p53-dependent pathway. Arterioscler Thromb Vasc Biol.

[59] Teodoro JG (2007). Inhibition of tumor angiogenesis by p53: a new role for the guardian of the genome. J Mol Med (Berl).

[60] Stone OA (2018). Loss of pyruvate kinase M2 limits growth and triggers innate immune signaling in endothelial cells. Nat Commun.

[61] Kim B (2018). Endothelial pyruvate kinase M2 maintains vascular integrity. J Clin Invest.

[62] Van der Veldt AA (2012). Rapid decrease in delivery of chemotherapy to tumors after anti-VEGF therapy: implications for scheduling of anti-angiogenic drugs. Cancer Cell.

[63] Schaaf MB (2019). Autophagy in endothelial cells and tumor angiogenesis. Cell Death Differ.

[64] Lloyd AC (2013). The regulation of cell size. Cell.

[65] Pan M (2021). The chemotherapeutic CX-5461 primarily targets TOP2B and exhibits selective activity in high-risk neuroblastoma. Nat Commun.

[66] Bergeron-Sandoval LP (2016). Mechanisms and consequences of macromolecular phase separation. Cell.

[67] Jin LH (2009). Requirement of Split ends for epigenetic regulation of Notch signal-dependent genes during infection-induced hemocyte differentiation. Mol Cell Biol.

[68] Girard V (2020). Spen modulates lipid droplet content in adult Drosophila glial cells and protects against paraquat toxicity. Sci Rep.

[69] Wang Y (2010). Ephrin-B2 controls VEGF-induced angiogenesis and lymphangiogenesis. Nature.

[70] Maeder ML (2013). CRISPR RNA-guided activation of endogenous human genes. Nat Methods.

[71] Leone S (2017). The RNA helicase DHX9 establishes nucleolar heterochromatin, and this activity is required for embryonic stem cell differentiation. EMBO Rep.

[72] Santoro R (2010). Intergenic transcripts originating from a subclass of ribosomal DNA repeats silence ribosomal RNA genes in trans. EMBO Rep.

[73] https://www.omicshare.com/tools.

[74] Donati G (2013). 5S ribosomal RNA is an essential component of a nascent ribosomal precursor complex that regulates the Hdm2-p53 checkpoint. Cell Rep.

