# Supplementary material for: Repression of rRNA gene transcription by endothelial SPEN deficiency normalizes tumor vasculature via nucleolar stress
Source: J Clin Invest. 2023 Oct 16;133(20):e159860. doi: 10.1172/JCI159860 (PMC10575731; doi:10.1172/JCI159860)
Supplement: Supplemental data [file jci-133-159860-s143.pdf]

Supplemental Materials

Repression of rRNA gene transcription by endothelial SPEN deficiency normalizes tumor vasculature via nucleolar stress

Zi-Yan Yang, Xian-Chun Yan, Jia-Yu-Lin Zhang, Liang Liang, Chun-Chen Gao, Pei-Ran Zhang, Yuan Liu, Jia-Xing Sun, Bai Ruan, Juan-Li Duan, Ruo-Nan Wang, Xing-Xing Feng, Bo Che, Tian Xiao, Hua Han

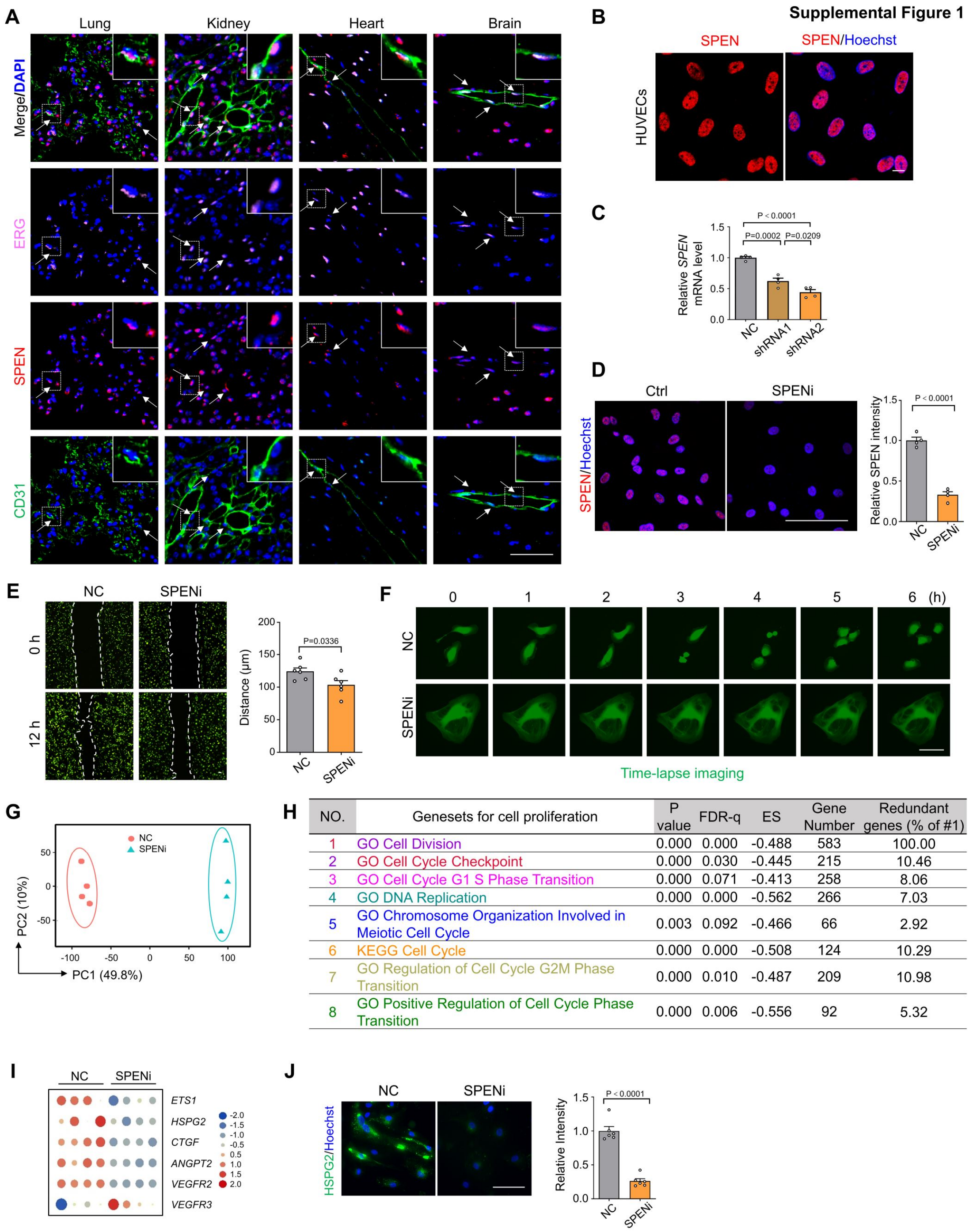

NO.	Genesets for cell proliferation	P value	FDR-q	ES	Gene Number	Redundant genes (% of #1)
1	GO Cell Division	0.000	0.000	-0.488	583	100.00
2	GO Cell Cycle Checkpoint	0.000	0.030	-0.445	215	10.46
3	GO Cell Cycle G1 S Phase Transition	0.000	0.071	-0.413	258	8.06
4	GO DNA Replication	0.000	0.000	-0.562	266	7.03
5	GO Chromosome Organization Involved in Meiotic Cell Cycle	0.003	0.092	-0.466	66	2.92
6	KEGG Cell Cycle	0.000	0.000	-0.508	124	10.29
7	GO Regulation of Cell Cycle G2M Phase Transition	0.000	0.010	-0.487	209	10.98
8	GO Positive Regulation of Cell Cycle Phase Transition	0.000	0.006	-0.556	92	5.32

Supplemental Figure 1. SPEN knockdown represses EC proliferation. (**A**) Sections of mouse lung, kidney, heart and brain were stained by SPEN, CD31, ERG immunofluorescence. White arrows indicate co-localizing signals. Scale bar, 50 μm. (**B**) HUVECs were stained by SPEN immunofluorescence. Scale bar, 10 μm. (**C**) HUVECs were transduced with NC or SPEN shRNAs lentivirus. The SPEN knockdown efficiency was determined by RT–qPCR (n = 4). (**D**) HUVECs were transduced with

NC or SPENi (shRNA2) lentivirus. SPEN knockdown efficiency was determined by immunofluorescence (n = 4). Scale bar, 100 μm. (**E**) HUVECs were transduced with NC or SPENi lentivirus expressing EGFP. Cell migration was analyzed by the wound-healing assay (n = 6). Scale bar, 100 μm. (**F**) HUVECs were transduced with NC or SPENi lentivirus expressing EGFP. Cells were recorded with a living cell imaging workstation and cell images on different time points were shown. (**G**) HUVECs transduced with NC or SPENi lentivirus were subjected to RNA-seq, and data were analyzed with PCA (n = 4 biological replicates). (**H**) List of gene sets for the GSEA of cell cycle pathways in HUVECs transduced with NC or SPENi lentivirus (Figure 1D). The number of genes in each gene set was listed, and the redundancy of genes among different gene sets was estimated by percentage of identical genes compared with the gene set #1. (**I**) Transcriptomes of HUVECs transduced with NC or SPENi lentivirus were analysed for genes associated with angiogenesis by Heatmap. (**J**) HUVECs were transduced with NC or SPENi lentivirus. The expression of HSPG2 was determined by immunofluorescence (n = 6). Scale bar, 100 μm. Data represent mean ± SEM; one-way ANOVA with Tukey's multiple comparisons test in (**C**), and unpaired two-sided Student’s t-test for others.

Supplemental Figure 2

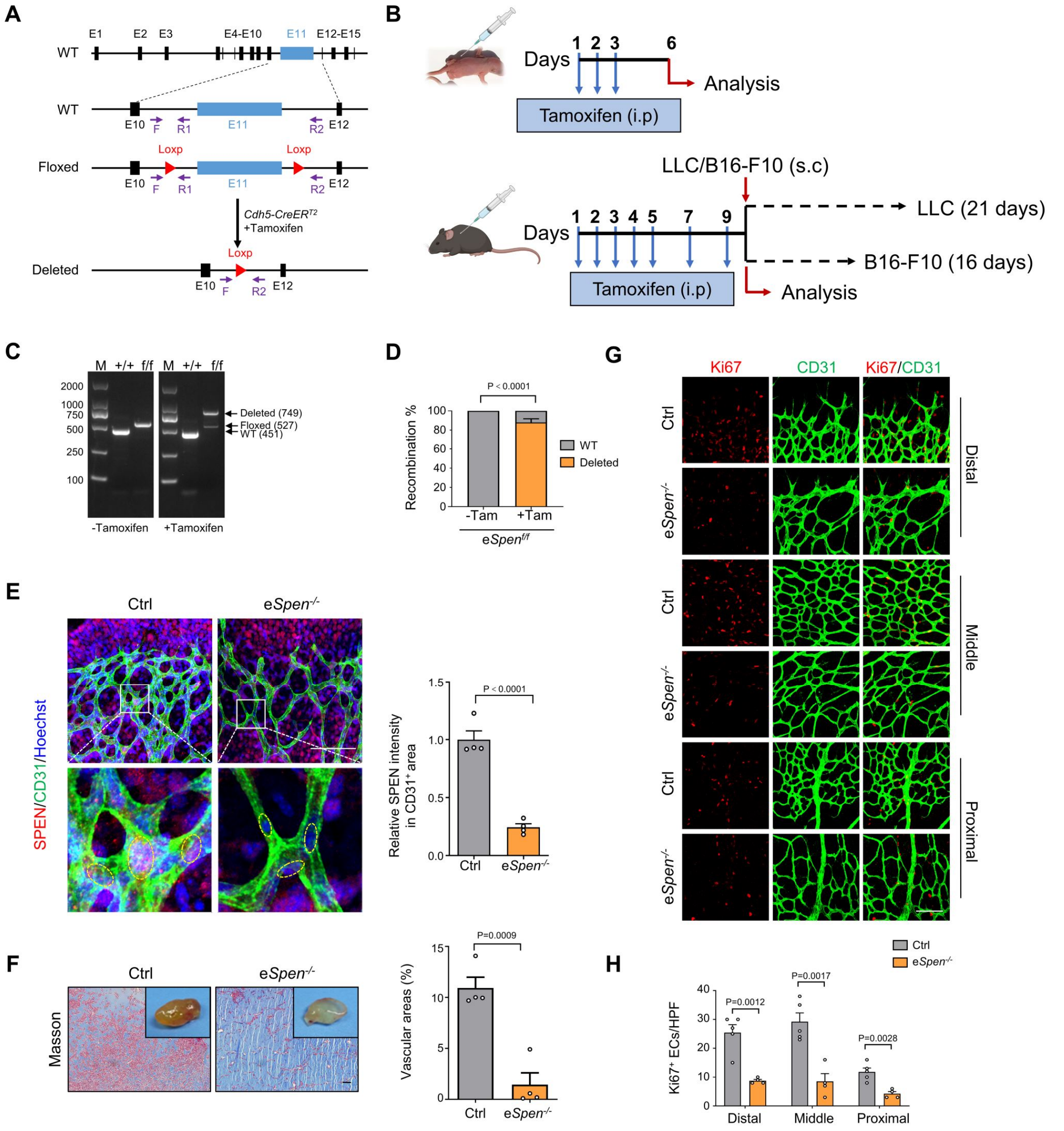

Supplemental Figure 2. Endothelial *Spen* ablation retards angiogenesis. (**A**–**E**) EC-specific *Spen* ablation in mice, as schematically shown in (**A**). *Cdh5-Cre*^{ERT2}*-Spen-floxed* (e*Spen*^{f}) mice were genotyped with their tail DNA and then induced with tamoxifen under different schedules (**B**). Brain ECs were isolated from adult e*Spen*$^{+/+}$ and e*Spen*$^{f/f}$ mice and subjected to PCR with EC genomic DNA as a template using primers F+R2 (deleted) or F+R1 (floxed or wild type) (**C**). The recombination efficiency (Deleted/[Deleted+Floxed] in e*Spen*$^{f/f}$ mice with or without tamoxifen induction) was determined by quantifying the amplified bands (**D**) (n = 5). In (**E**), retinas from P6 control and e*Spen*$^{-/-}$ mice were subjected to immunofluorescence, and

SPEN protein level in EC nuclei (marked with yellow dashed circles) was quantitatively compared (n = 4). Scale bar, 100 μm. (**F**) Pro-angiogenic Matrigel plugs were embedded in mice. The plugs were recovered 7 days later, photographed and subjected to Masson's staining. The vascular areas were quantified (n = 4). Scale bar, 100 μm. (**G** and **H**) Whole-mount immunofluorescence staining of retinas from Ctrl and e*Spen*$^{-/-}$ mice with Ki67 and CD31. The Ki67^{+} ECs in different angiogenic zones of retinas were compared (**H**) (n = 5 and 4 for Ctrl and e*Spen*$^{-/-}$, respectively). Scale bar, 100 μm. Data represent mean ± SEM; unpaired two-sided Student's t-test.

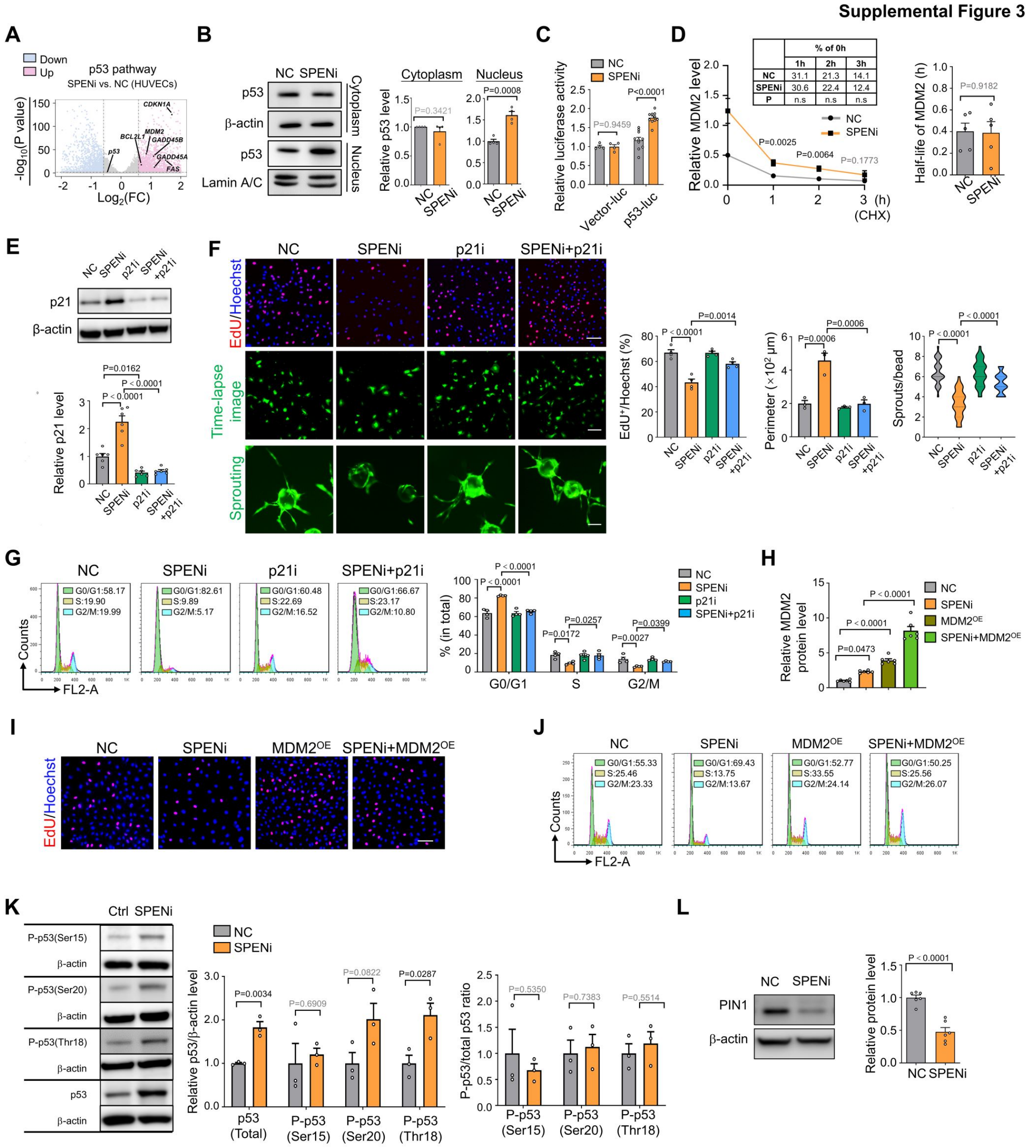

Supplemental Figure 3. SPEN knockdown represses EC proliferation via the p53-p21 signaling. (**A**) The RNA-seq data of HUVECs transduced with NC or SPENi lentivirus (Supplemental Figure 1G) are shown by the volcano plot, and p53 downstream genes are indicated. (**B**) HUVECs were transduced with NC or SPENi lentivirus. The p53 level in nuclear and cytoplasmic fractions was determined by immunoblotting (n = 4). (**C**) HEK293T cells were transduced with NC or SPENi lentivirus and the p53 reporter plasmid (p53-luc). Luciferase activity was determined 24 h after the reporter transfection (n = 4 for Vector-luc and n = 10 for p53-luc). (**D**) The MDM2 level in Figure 2D was plotted and its half-life was determined (n = 5). The

inset table shows the percentage of MDM2 level at different time points vs MDM2 level of 0 h after CHX addition (n.s, not significant). (**E**–**G**) HUVECs were transduced with NC, SPENi, p21i, or SPENi+p21i lentivirus expressing EGFP, and p21 level was assessed by immunoblotting (**E**) (n = 6). The cells were subjected to the EdU incorporation assay, live cell imaging, and microbead sprouting assay in (**F**), and the cell proliferation (n = 4), cell size (n = 3), and sprouts (n = 30 beads from 3 biological replicates) were quantified. The cells were subjected to cell cycle analysis in (**G**), and the cell cycle distribution was quantitatively compared (n = 4). (**H**–**J**) HUVECs were transduced with SPENi or NC lentivirus, and simultaneously transduced with MDM2-overexpressing lentivirus. MDM2 level (**H**) (n = 6), cell proliferation (**I**) (n = 6) and cell cycle progression (**J**) (n = 3) were analysed. Scale bar, 100 μm. (**K** and **L**) HUVECs were transduced with SPENi or NC, and total p53 and phosphorylated p53 levels (**K**) (n = 3), as well as the level of PIN1 (**L**) (n = 6), were determined by immunoblotting. Scale bars, 100 μm. Data represent mean ± SEM; unpaired two-sided Student’s t-test in (**B**–**D**, **K** and **L**), and one-way ANOVA with Tukey's multiple comparisons test in (**E**–**J**).

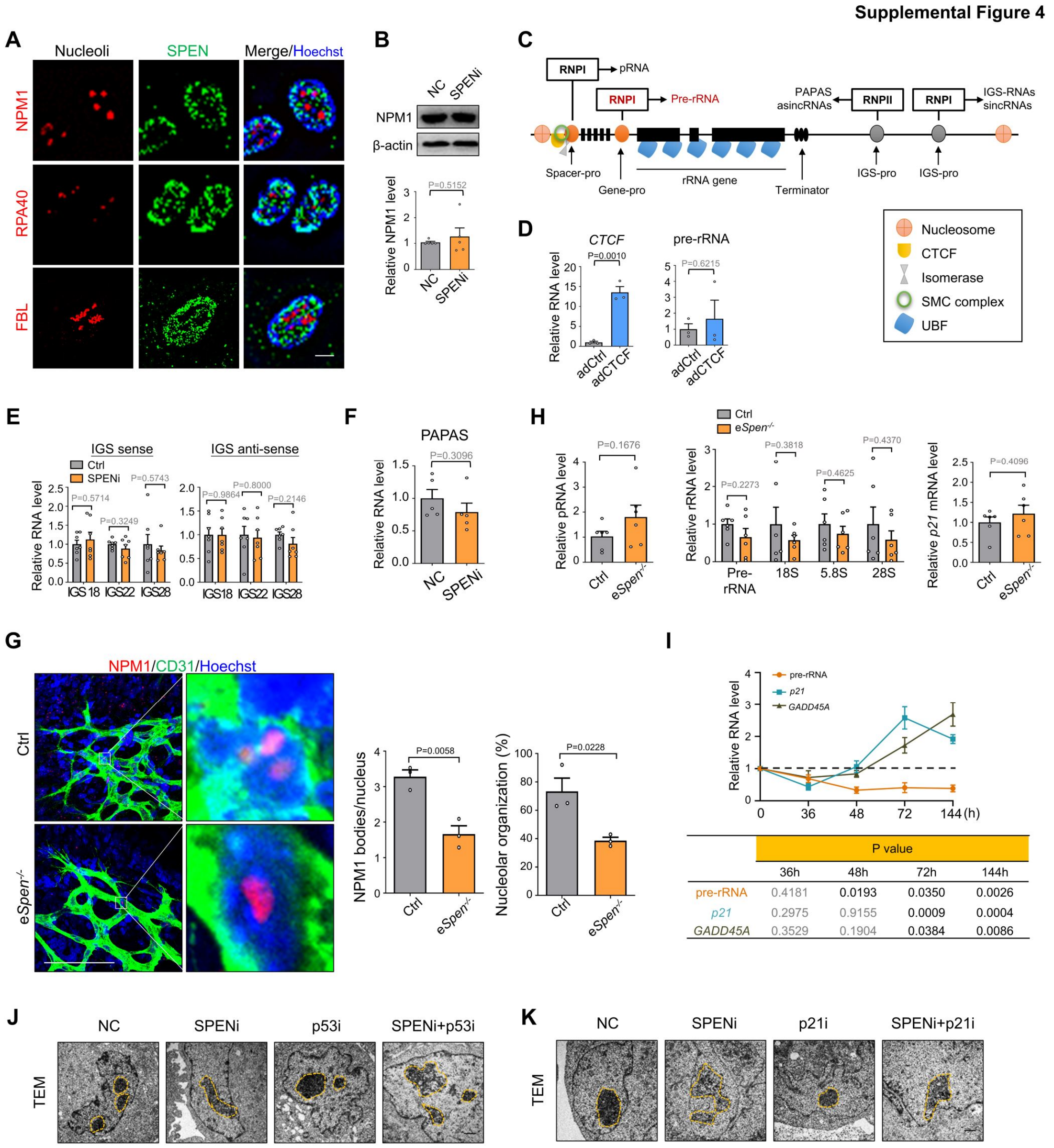

Supplemental Figure 4. SPEN knockdown triggers nucleolar stress in ECs. (**A**) HUVECs were stained by immunofluorescence with anti-SPEN together with anti-NPM1, anti-RPA40, or anti-FBL and analyzed by SIM microscopy. Scale bar, 5 μm. (**B**) NPM1 expression in HUVECs transduced with NC or SPENi lentivirus as determined by immunoblotting (n = 5). (**C**) Schematic structure of the human genomic rDNA unit. (**D**) HUVECs were transduced with Ctrl or CTCF adenovirus. The expression of *CTCF* and pre-rRNA was determined by RT–qPCR (n = 3). (**E**) HUVECs were transduced with NC or SPENi lentivirus. The expression of sense and antisense IGS RNAs was determined by strand-specific RT–qPCR (n = 7). (**F**) Expression of lncRNA PAPAS in

HUVECs transduced with NC or SPENi lentivirus as determined by strand-specific RT–qPCR (n = 5). (**G**) Whole mount retinal samples from P6 e*Spen*$^{-/-}$ and control pups were stained by CD31 and NPM1 immunofluorescence, and observed under a laser scanning confocal microscope. The nucleolar bodies per nuclei and ECs containing nucleoli with normal morphology were quantitatively compared (n = 3). Scale bar, 100 μm. (**H**) ECs were isolated from the brain of adult e*Spen*$^{-/-}$ and control mice, and the expression of pRNA, pre-rRNA and mature rRNA, as well as *p21* was determined by RT-qPCR (n = 6). (**I**) HUVECs were transduced with NC or SPENi lentivirus. The expression of pre-rRNA, *p21*, and *GADD45A* was determined by RT–qPCR at 36 (n = 3), 48 (n = 6), 72 (n= 5, 6 and 6 for pre-rRNA, *p21*, and *GADD45A*, respectively) and 144 h (n= 6, 4 and 4 for pre-rRNA, *p21*, and *GADD45A*, respectively). The dotted line represents the expression level in control groups. (**J** and **K**) HUVECs were transfected as indicated and observed under TEM (nucleoli, yellow dashed lines). Scale bars, 1 μm. Data represent mean ± SEM; unpaired two-sided Student's t-test.

Supplemental Figure 5

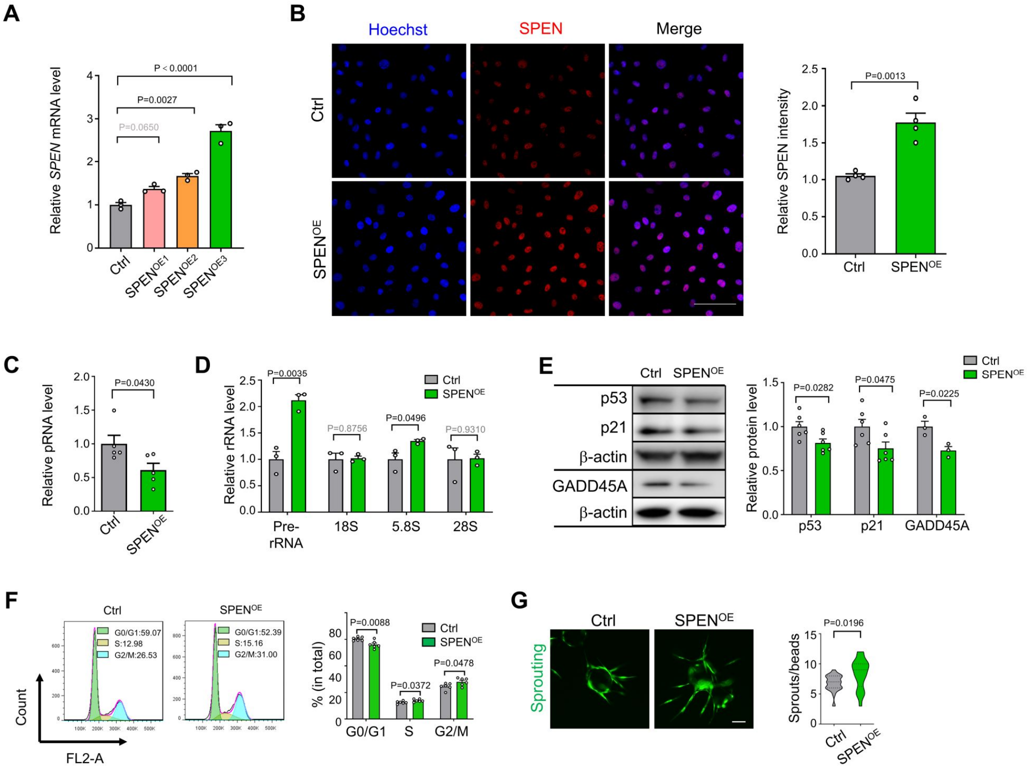

Supplemental Figure 5. SPEN upregulation represses pRNA and promotes ribosomal gene expression. (**A, B**) HUVECs were transduced with lenti-dCAS9-VP64-Puro and lenti-sgRNA-MS2-P65-HSF1-Neo, and the *SPEN* mRNA level was determined by RT-qPCR (n = 3). In (**B**), cells transfected with the $SPEN^{OE3}$ was stained by SPEN immunofluorescence and quantified (n = 4). Scale bar, 100 µm. (**C–E**) The expression of pRNA (n = 5), pre-rRNA and mature rRNA (n = 3) was determined by RT–qPCR, and the expression of p53 and its downstream molecules p21 and GADD45A was determined by immunoblotting (n = 6, 6, and 3 for p53, p21, and GADD45A, respectively). (**F**) Cell cycle analysis (n = 6). (**G**) Sprouting assay (n = 20 beads from 3 biological replicates). Scale bar, 100 µm. Data represent mean ± SEM; unpaired two-sided Student's t-test in (**B–G**), and one-way ANOVA with Tukey's multiple comparisons test in (**A**).

Supplemental Figure 6

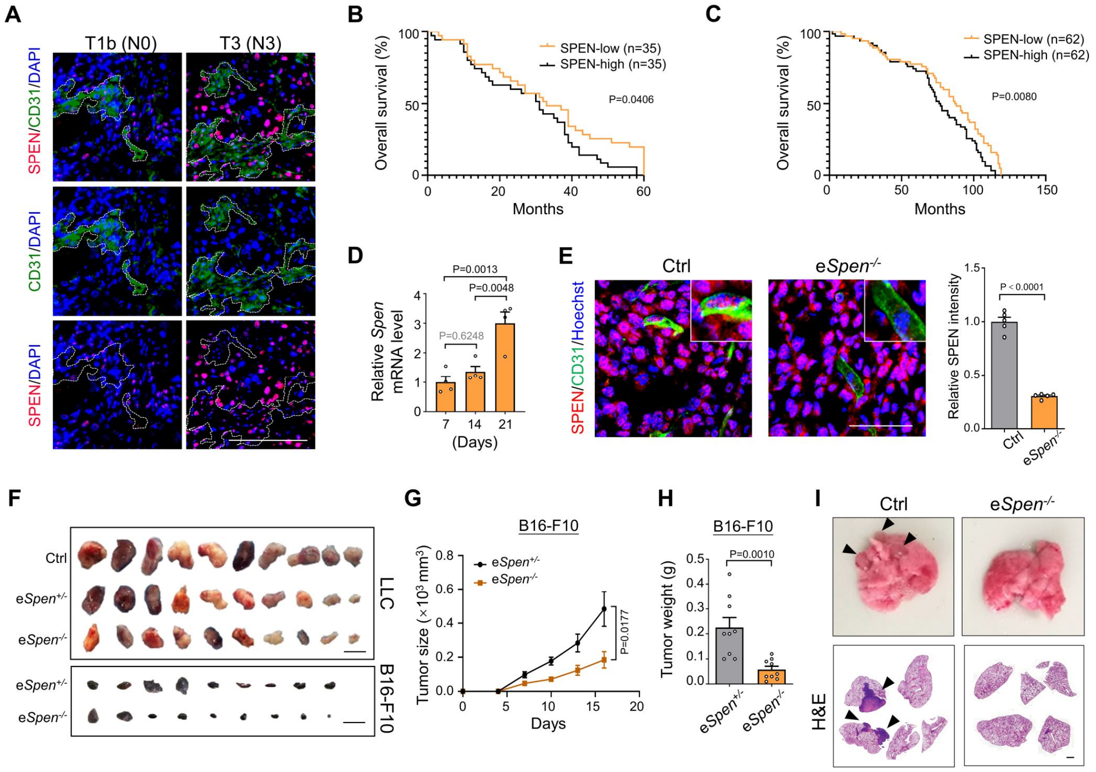

Supplemental Figure 6. Endothelial *Spen* ablation represses tumor growth and metastasis. (**A**) Human lung cancer biopsies were immunostained for CD31 and SPEN. Scale bar, 100 µm. (**B, C**) Gastric cancer and breast cancer samples were stained for CD31 and SPEN, and analyzed for the correlation of endothelial SPEN level and prognosis. n = 35 patients per group for gastric cancer samples, and n = 62 patients per group for breast cancer samples. (**D**) TECs were isolated from C57BL/6J mice inoculated with LLC cells at 7, 14, and 21 dpi. The expression of *Spen* was determined by RT-qPCR (n = 4). (**E**) Mice with different genotypes were inoculated with LLC. Tumor sections were stained by immunofluorescence to evaluate Spen ablation efficiency (n = 5). Scale bar, 50 µm. (**F**) Mice with different genotypes were inoculated with LLC or B16-F10 cells. LLC tumors were dissected on 21 dpi, and B16-F10 tumors were dissected on 16 dp. Tumors were photographed. Scale bar, 1 cm. (**G** and **H**) Mice with different genotypes were inoculated with B16-F10 cells. Tumor sizes were monitored and tumor weights were compared on 16 dpi (n = 9). (**I**) The LLC tumors in Ctrl and e*Spen*$^{-/-}$ mice were removed on 14 dpi, and the mice were maintained for 28 more days. Lung samples were obtained, photographed, and stained with H&E. The

arrowheads indicate metastatic tumors. Scale bar, 1 mm for H&E. Data represent mean ± SEM; unpaired two-sided Student's t-test except for log-rank (Mantel-Cox) test in (**B** and **C**).

Supplemental Figure 7

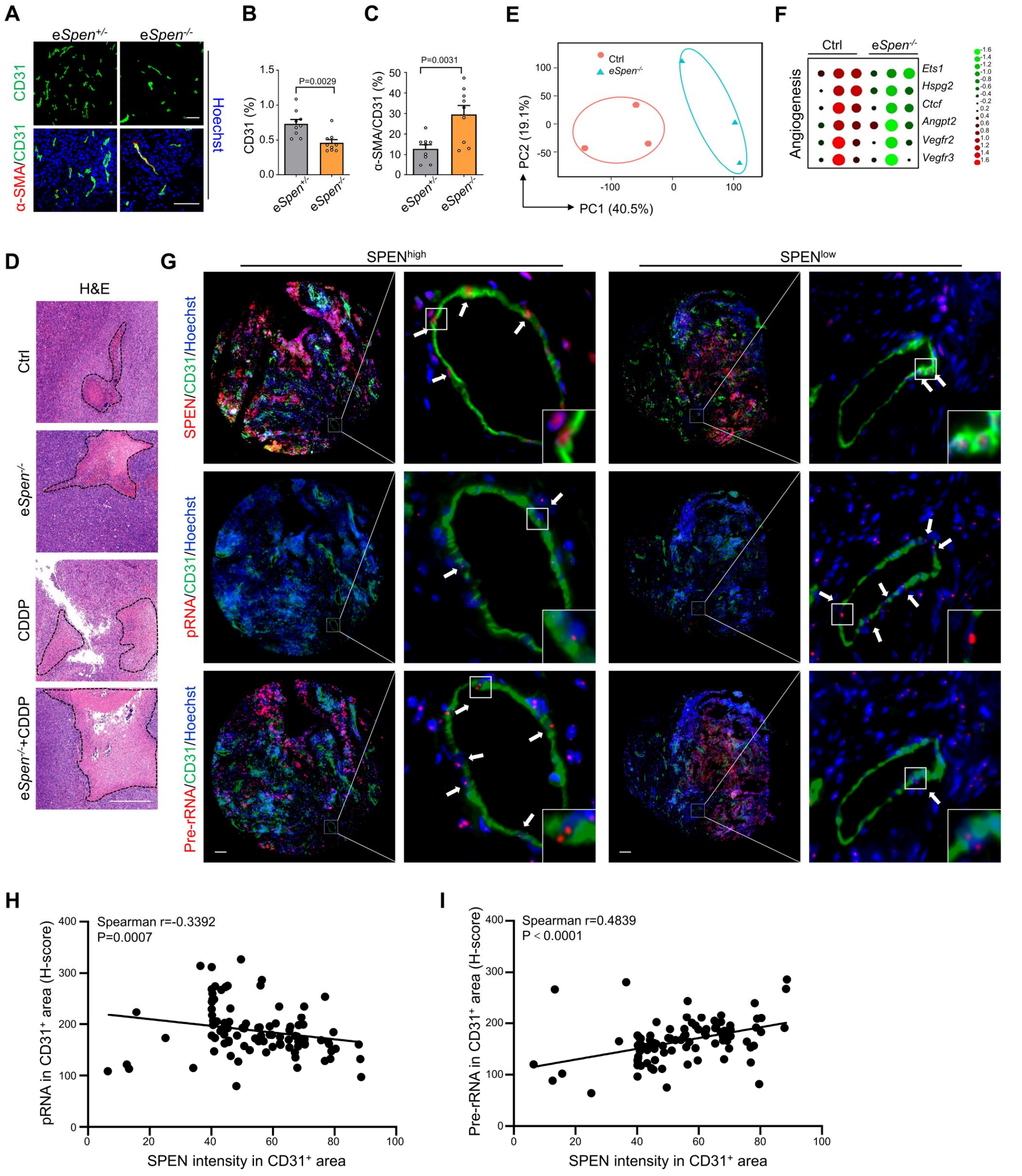

Supplemental Figure 7. Endothelial SPEN deficiency normalizes tumor vessels. (**A**–**C**) e*Spen*$^{+/-}$ and e*Spen*$^{-/-}$ mice were inoculated with B16-F10 cells. Tumors were dissected on 16 dpi and stained by immunofluorescence. The vessel density (CD31^{+}) and pericyte coverage (α-SMA^{+}/CD31^{+}) were quantified (n = 9). Scale bars, 100 μm. (**D**) Mice bearing LLC tumors were treated with CDDP from 7 dpi. Tumor sections were stained with H&E. Dotted lines, necrosis areas. Scale bar, 500 μm. (**E**) PCA was used to cluster the RNA-seq data from Ctrl and e*Spen*$^{-/-}$ TECs. (**F**) The angiogenesis-related genes in Ctrl and e*Spen*$^{-/-}$ TECs are shown in a heatmap. (**G**–**I**) Human lung

cancer biopsies were serially sectioned, and stained by immunofluorescence for CD31 and SPEN, and simultaneously stained by in situ hybridization to detect pRNA or pre-rRNA. ECs ($CD31^+$) were divided into $SPEN^{high}$ and $SPEN^{low}$ groups, and the correlation between SPEN level and pRNA or pre-rRNA level was determined (n = 96). Scale bar, 100 μm. Data represent mean ± SEM; unpaired two-sided Student's t-test in (**B** and **C**), and Spearman's rank-order correlation analysis in (**H** and **I**).

Supplemental Figure 8

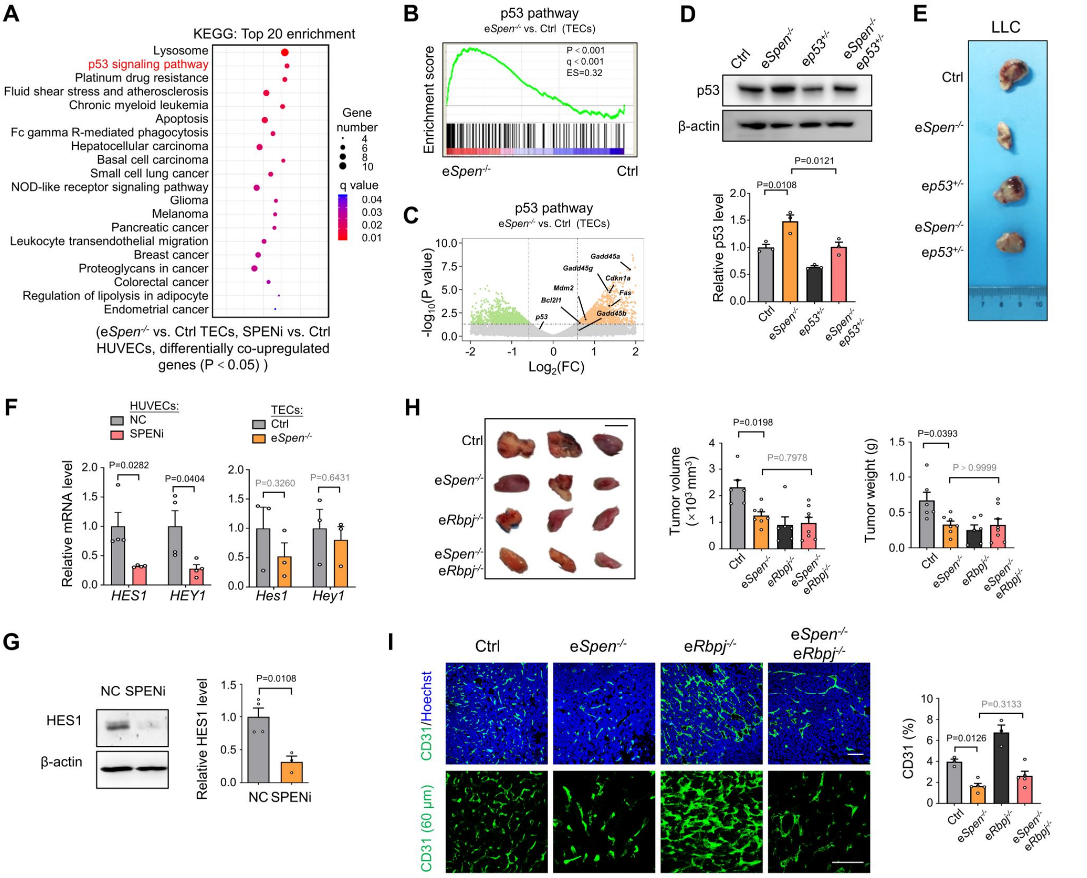

Supplemental Figure 8. SPEN deficiency-induced tumor vessel normalization is dependent on p53 but not Notch activation. (**A**) KEGG analysis of differentially co-upregulated genes in transcriptomic data, and the top 20 significantly changed entries were presented. (**B** and **C**) Profiling of p53-related genes in Ctrl and e*Spen*$^{-/-}$ TECs by GSEA (**B**). In (**C**), some of the p53 downstream genes are highlighted. (**D**) Mice with different genotypes (Ctrl, e*Spen*$^{-/-}$, e*p53*$^{+/-}$, and e*Spen*$^{-/-}$e*p53*$^{+/-}$) were inoculated with LLC cells. Tumor ECs were isolated on 21 dpi, and p53 level was determined by immunoblotting (n = 3). (**E**) Mice with different genotypes were inoculated with LLC, and tumors were dissected on 21 dpi and photographed. (**F**) Expression of *HES1* and *HEY1* in HUVECs transduced with NC or SPENi lentivirus as well as in e*Spen*$^{-/-}$ and Ctrl TECs was determined by RT–qPCR (n = 4 and 3 for HUVECs and TECs, respectively). (**G**) Expression of HES1 in HUVECs transduced with NC or SPENi lentivirus was determined by immunoblotting (n = 4 and 3 for NC and SPENi, respectively). (**H**) Mice were bred to obtain the indicated genotypes and inoculated with

LLC cells. Tumors were dissected on 21 dpi and photographed. The tumor sizes and weights were compared (n = 6, 7, 6 and 8 for Ctrl, e*Spen*$^{-/-}$, e*Rbpj*$^{-/-}$, and e*Spen*$^{-/-}$e*Rbpj*$^{-/-}$, respectively). Scale bar, 1 cm. (**I**) Tumor sections were stained by immunofluorescence, and the vessel density ($CD31^{+}$) was compared (n = 3, 5, 3 and 5 for Ctrl, e*Spen*$^{-/-}$, e*Rbpj*$^{-/-}$, *and* e*Spen*$^{-/-}$e*Rbpj*$^{-/-}$, respectively). Scale bars, 100 µm. Data represent mean ± SEM; unpaired two-sided Student's t-test in (**F**, **G**); one-way ANOVA with Tukey's multiple comparisons test in (**D**, **H** and **I**).

Supplemental Figure 9

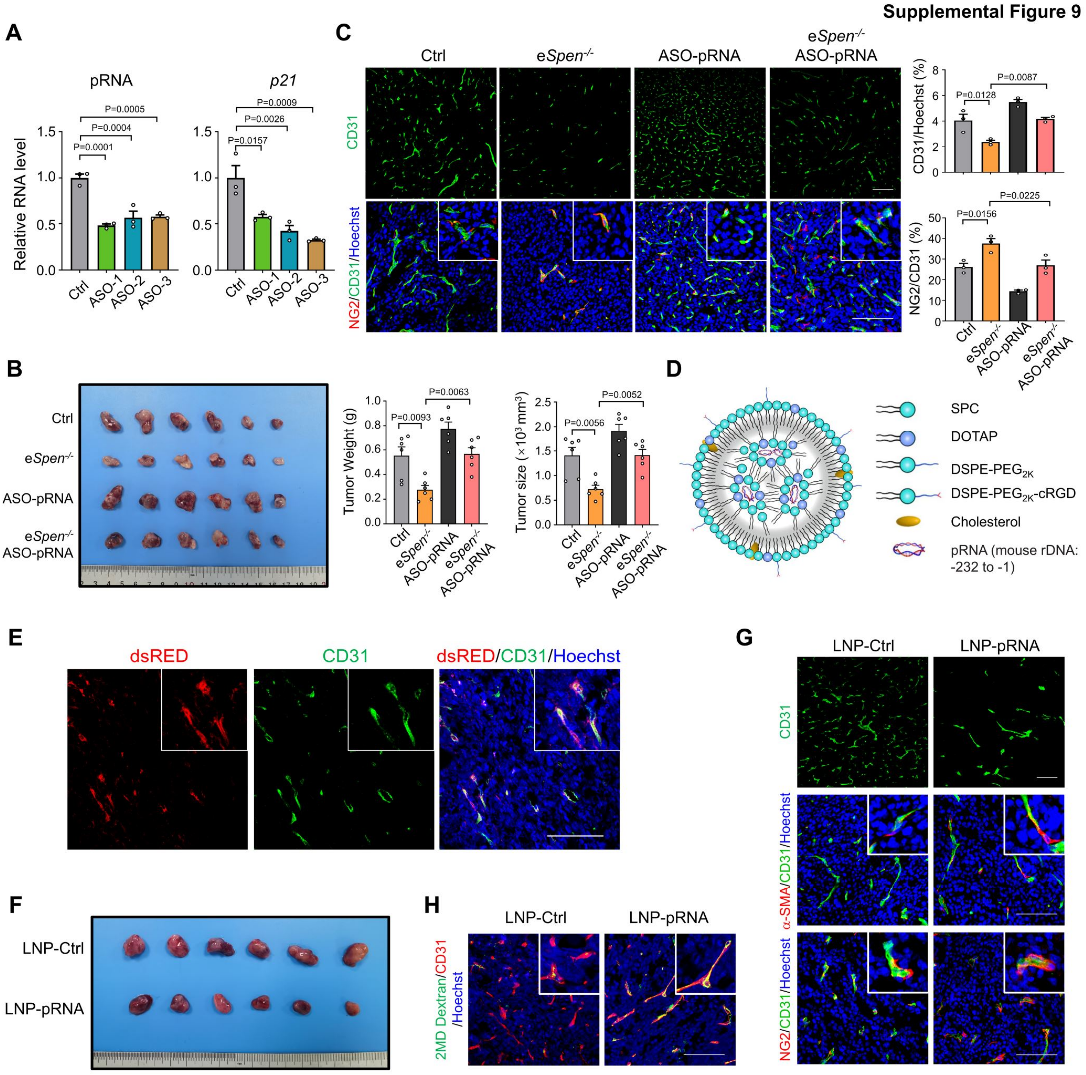

Supplemental Figure 9. pRNA regulates tumor vessels *in vivo*. (**A**) The bEND.3 EC line was transfected with ASO-pRNAs, and the level of pRNA and *p21* was determined by RT–qPCR (n = 3). (**B**, **C**) The e*Spen*$^{-/-}$ and Ctrl mice were inoculated with LLC, and ASO-pRNA or control was injected intra-tumorally from 10 dpi. Tumor growth (n = 6) and tumor vessels (n = 3) were examined as above. Scale bars, 100 μm. (**D**) Schematic illustration of the LNP expressing pRNA. (**E**) Liposome nanoparticles were loaded with a plasmid expressing dsRED, and injected i.v into tumor-bearing mice. The uptake of LNPs was determined under a laser scanning confocal microscope after CD31 staining (green). (**F**–**H**) Tumor-bearing mice were infused with LNP-pRNA or LNP-Ctrl. Tumor growth was evaluated (**F**), tumor vessels were stained with immunofluorescence (**G**), and tumor vessel perfusion was evaluated using FITC-Dextran-2MD (**H**). Scale

bars, 100 μm. Data represent mean ± SEM; one-way ANOVA with Tukey's multiple comparisons test.

Supplemental Figure 10

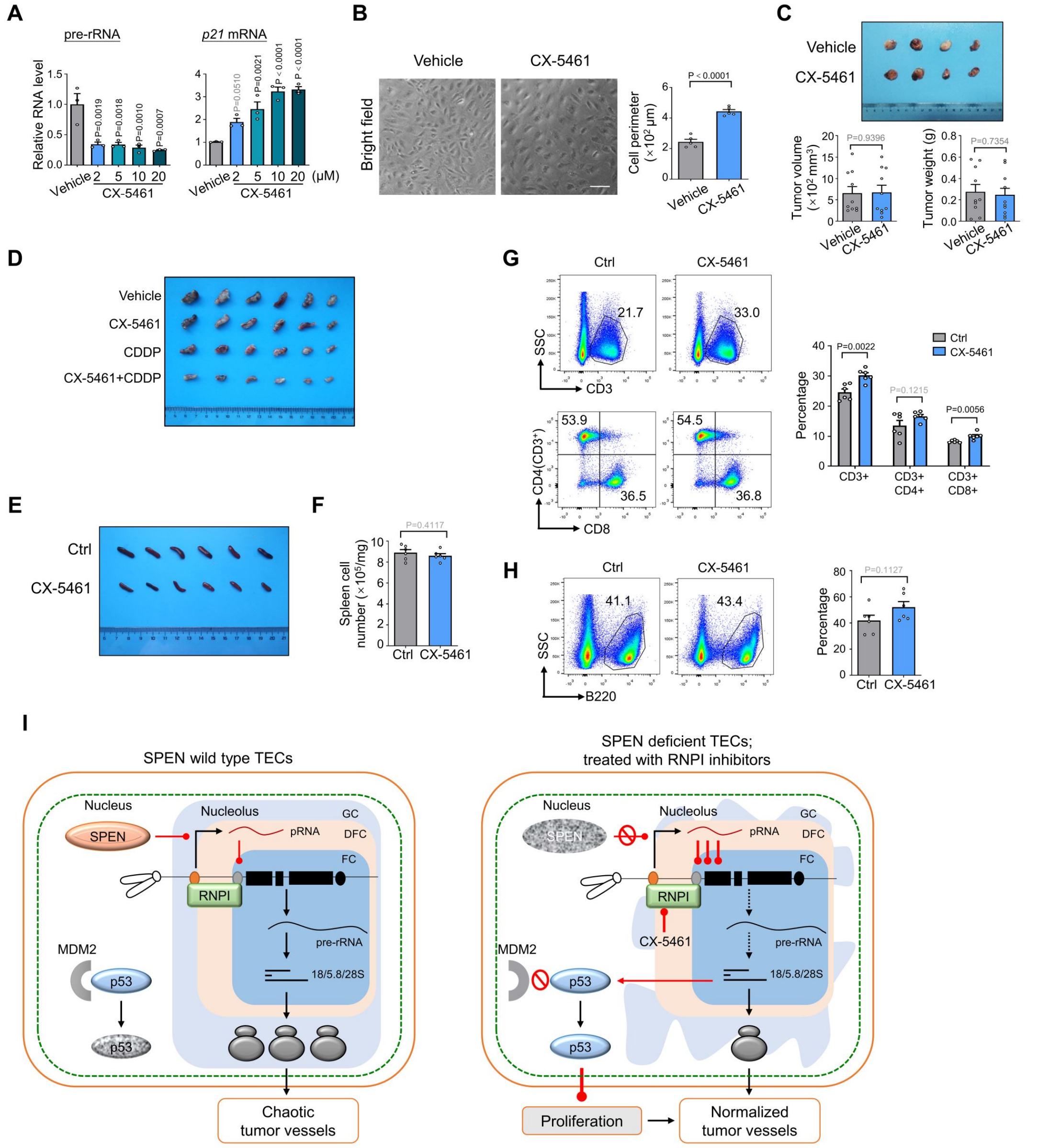

Supplemental Figure 10. CX-5461, an RNPI inhibitor, induces tumor vessel normalization. (**A**) HUVECs were treated with vehicle or CX-5461 for 48 h. The expression of pre-rRNA and *p21* was determined by RT–qPCR (n = 3). (**B**) HUVECs were treated with vehicle or 2 μM CX-5461 for 48 h and photographed. The cell perimeter was assessed (n = 5). Scale bar, 100 μm. (**C**) Tumor-bearing mice were orally administered with CX-5461. The tumors were dissected and photographed. The tumor sizes and weights were compared on 14 dpi (n = 10). (**D**) Mice bearing LLC tumors were orally administered with 50 mg/kg CX-5461 every two days and injected i.p with CDDP every three days from 7 to 14 dpi. The tumors were dissected and photographed

on 14 dpi. (**E**–**H**) Spleens were collected from LLC-bearing mice treated with CX-5461, photographed, and analyzed by FACS for T and B lymphocytes after staining with different combinations of antibodies (n = 6). (**I**) Schematic illustration showing the role and mechanism of SPEN and RNPI inhibitors in regulating tumor angiogenesis. See text for details. Data represent mean ± SEM; unpaired two-sided Student's t-test except for one-way ANOVA with Tukey's multiple comparisons test in (**A**).

Supplemental Table 1. Information of patients enrolled in the human lung adenocarcinoma tissue microarray (HLugA180Su07, Outdo Biotech).

NO.	Sex	Age	Number of metastasis positive lymph nodes	T	N	M	AJCC stage	Survival (months)
1	Female	59	14	T3	N1	M0	3A	38
2	Male	49	0	T1b	N0	M0	1A	91
3	Female	53	0	T1b	N0	M0	1A	88
4	Male	74	0	T2a	N0	M0	1B	21
5	Male	58	0	T2a	N0	M0	1B	39
6	Male	30	0	T1b	N0	M0	1A	34
7	Female	64	0	T2a	N0	M0	1B	15
8	Female	50	4	T3	N1	M0	3A	55
9	Female	46	3	T3	N1	M0	3A	10
10	Male	47	1	T3	N1	M0	3A	33
11	Male	65	15	T3	N2	M0	3A	14
12	Female	58	0	T2a	N0	M1b	4	49
13	Female	67	1	T2a	N1	M0	2A	13
14	Female	50	0	T1b	N0	M0	1A	67
15	Female	76	0	T1b	N0	M0	1A	15
16	Female	62	11	T3	N3	M0	3B	9
17	Male	74	2	T3	N1	M0	3A	10
18	Male	49	4	T2a	N2	M0	3A	17
19	Male	73	3	T2b	N1	M0	2B	33
20	Male	75	1	T2a	N1	M0	2A	59
21	Male	75	0	T4	N0	M0	3A	27
22	Female	52	12	T3	N3	M0	3B	44
23	Male	65	2	T2a	N1	M0	2A	25
24	Male	74	0	T2a	N0	M0	1B	56
25	Male	60	0	T2a	N0	M0	1B	62
26	Female	51	3	T1a	N2	M0	3A	29
27	Male	53	5	T2a	N2	M0	3A	16
28	Female	65	0	T2a	N0	M0	1B	14
29	Male	71	4	T3	N1	M0	3A	33
30	Female	60	8	T3	N3	M0	3B	40
31	Male	61	9	T2b	N3	M0	3B	15
32	Female	58	0	T3	N0	M0	2B	55
33	Female	58	0	T3	N0	M0	2B	35
34	Male	60	1	T3	N1	M0	3A	54
35	Male	63	3	T3	N2	M0	3A	25
36	Male	63	3	T2a	N1	M0	2A	49
37	Male	61	0	T2a	N0	M0	1B	39
38	Female	81	1	T2a	N1	M0	2A	52
39	Male	61	0	T2b	N0	M0	2A	7
40	Male	65	6	T2a	N2	M0	3A	50
41	Male	64	1	T2a	N1	M0	2A	49
42	Male	53	0	T2a	N0	M0	1B	50
43	Female	73	4	T4	N2	M0	3B	49
44	Male	52	6	T2a	N2	M0	3A	14
45	Male	55	7	T2b	N2	M0	3A	12

46	Female	50	0	T1a	N0	M0	1A		15
47	Female	60	0	T2b	N0	M0	2A		2
48	Male	54	3	T4	N2	M0	3B		2
49	Female	54	2	T3	N1	M0	3A		29
50	Male	59	10	T2a	N3	M0	3B		2
51	Male	78	0	T1b	N0	M0	1A		39
52	Male	58	0	T2a	N0	M0	1B		43
53	Female	56	4	T4	N2	M0	3B		15
54	Female	53	0	T2a	N0	M0	1B		42
55	Male	72	11	T2a	N2	M0	3A		30
56	Male	84	0	T2b	N0	M0	2A		24
57	Female	65	0	T2a	N0	M0	1B		40
58	Male	65	2	T3	N2	M0	3A		8
59	Female	77	8	T3	N3	M0	3B		29
60	Female	66	0	T2a	N0	M0	1B		37

Supplemental Table 2. Information of patients enrolled in the human lung adenocarcinoma tissue microarray (HLugA180Su08, Outdo Biotech).

NO.	Sex	Age	Number of metastasis positive lymph nodes	T	N	M	AJCC stage	Survival (months)
1	Male	47	10	T2a	N1	M0	2A	94
2	Female	72	0	T1b	N0	M0	1A	78
3	Female	66	9	T2a	Nx	M0	2-3	49
4	Male	60	0	T2b	N0	M0	2A	91
5	Male	49	0	T1b	N0	M0	1A	91
6	Male	66	0	T3	N0	M0	2B	90
7	Female	53	0	T1b	N0	M0	1A	88
8	Male	68	0	——	N0	M0	1-2	88
9	Male	74	1	T2a	Nx	M0	2-3	33
10	Male	58	0	T2a	N0	M0	1B	39
11	Male	30	0	T1b	N0	M0	1A	34
12	Male	67	14	T2b	Nx	M0	2-3	39
13	Male	57	0	T2a	N0	M0	1B	79
14	Female	25	1	——	Nx	M0	2-3	78
15	Female	64	0	T2a	N0	M0	1B	15
16	Male	50	0	T2a	N0	M0	1B	73
17	Male	57	0	T1b	N0	M0	1A	71
18	Female	46	3	T3	N1	M0	3A	10
19	Female	55	0	T1b	N0	M0	1A	71
20	Male	60	0	T2a	N0	M0	1B	62
21	Male	47	1	T3	N1	M0	3A	33
22	Male	65	15	T3	N2	M0	3A	14
23	Female	58	0	T2a	N0	M1b	4	49
24	Female	67	1	T2a	N1	M0	2A	13
25	Female	40	0	——	N0	M0	1-2	68
26	Female	50	0	T1b	N0	M0	1A	67
27	Female	68	0	T1b	N0	M0	1A	66
28	Male	55	0	T2a	N0	M0	1B	66
29	Female	56	0	T1	N0	M0	1A	66
30	Female	62	11	T3	N3	M0	3B	9
31	Male	74	2	T3	N1	M0	3A	10
32	Female	76	0	T1b	N0	M0	1A	15
33	Male	49	4	T2a	N2	M0	3A	17
34	Male	75	1	T2a	N1	M0	2A	59
35	Female	52	12	T3	N3	M0	3B	44
36	Male	65	2	T2a	N1	M0	2A	25
37	Male	45	0	T2a	N0	M0	1B	62
38	Male	59	0	T2a	N0	M0	1B	62
39	Male	64	7	T2	N2	M0	3A	62
40	Male	42	1	T1b	Nx	M0	2-3	13
41	Male	53	5	T2a	N2	M0	3A	16
42	Male	66	3	T2a	N1	M0	2A	6
43	Female	57	2	T2a	Nx	M0	2-3	40
44	Female	51	6	T2a	Nx	M0	2-3	57

45	Female	65	0	T2a	N0	M0	1B	14
46	Male	64	2	T2a	N1	M0	2A	58
47	Male	71	4	T3	N1	M0	3A	33
48	Female	60	8	T3	N3	M0	3B	40
49	Male	61	9	T2b	N3	M0	3B	15
50	Male	60	1	T3	N1	M0	3A	54
51	Male	63	3	T3	N2	M0	3A	25
52	Male	63	3	T2a	N1	M0	2A	49
53	Male	61	0	T2a	N0	M0	1B	39
54	Female	81	1	T2a	N1	M0	2A	52
55	Male	61	0	T2b	N0	M0	2A	7
56	Male	84	0	T2b	N0	M0	2A	24
57	Male	65	6	T2a	N2	M0	3A	50
58	Male	53	0	T2a	N0	M0	1B	50
59	Male	74	——	T1a	——	M0	——	1
60	Male	64	1	T2a	N1	M0	2A	49
61	Female	73	4	T4	N2	M0	3B	49
62	Male	52	6	T2a	N2	M0	3A	14
63	Male	44	1	T3	Nx	M0	3	3
64	Male	55	7	T2b	N2	M0	3A	12
65	Female	50	0	T1a	N0	M0	1A	15
66	Male	78	0	T1b	N0	M0	1A	39
67	Male	54	3	T4	N2	M0	3B	2
68	Female	54	2	T3	N1	M0	3A	29
69	Female	48	0	T3	N0	M0	2B	43
70	Male	59	21	T2a	Nx	M0	2-3	25
71	Male	58	0	T2a	N0	M0	1B	43
72	Female	56	4	T4	N2	M0	3B	15
73	Female	53	0	T2a	N0	M0	1B	42
74	Female	62	1	T1b	Nx	M0	2-3	10
75	Male	72	11	T2a	N2	M0	3A	30
76	Male	61	0	T2b	N0	M0	2A	40
77	Female	65	0	T2a	N0	M0	1B	40
78	Female	67	10	T1b	Nx	M0	2-3	39
79	Male	65	0	T3	N0	M0	2B	39
80	Female	66	0	T2a	N0	M0	1B	37
81	Male	74	0	T2a	N0	M0	1B	21
82	Female	20	0	T1b	N0	M0	1A	82
83	Male	51	2	——	Nx	M0	2-3	69
84	Male	73	3	T2b	N1	M0	2B	33
85	Female	57	10	T4	N2	M0	3B	3
86	Male	75	0	T4	N0	M0	3A	27
87	Male	60	0	T2a	N0	M0	1B	62
88	Male	74	0	T2a	N0	M0	1B	56
89	Female	51	3	T1a	N2	M0	3A	29
90	Female	71	0	T1b	N0	M0	1A	59
91	Female	58	0	T3	N0	M0	2B	55
92	Female	62	9	T1a	Nx	M0	2-3	55
93	Female	73	10	T2b	Nx	M0	2-3	12

94	Male	59	10	T2a	N3	M0	3B	2
95	Male	65	2	T3	N2	M0	3A	8
96	Female	77	8	T3	N3	M0	3B	29

Supplemental Table 3. Information of patients enrolled in the human gastric cancer tissue microarray (HStmA180Su30, Outdo Biotech).

NO.	Sex	Age	T	N	M	Survival (months)
1	Male	67	T4	N3	M0	37
2	Male	57	T3	N1	M0	10
3	Male	43	T3	N0	M0	60
4	Female	65	T4a	N0	M0	39
5	Male	70	T4	N2	M0	18
6	Male	53	T3	N0	M0	23
7	Male	67	T3	N3	M1	51
8	Female	69	T3	N2	M0	43
9	Male	75	T3	N0	M0	39
10	Female	64	T3	N0	M0	56
11	Male	41	T3	N2	M1	25
12	Male	50	T3	N3	M0	27
13	Female	60	T4	N3	M1	20
14	Female	68	T3	N0	M0	39
15	Female	51	T4	N3	M1	11
16	Male	69	T4	N3	M0	11
17	Male	69	T3	N2	M0	31
18	Female	59	T2	N3	M0	60
19	Male	69	T3	N2	M0	60
20	Male	64	T2	N0	M0	60
21	Male	56	T4	N3	M1	12
22	Male	40	T3	N2	M0	27
23	Male	70	T4	N2	M0	11
24	Male	62	T4	N3	M1	41
25	Female	76	T1b	N2	M0	32
26	Female	56	T4	N3	M1	13
27	Male	68	T3	N0	M0	60
28	Male	43	T3	N2	M0	60
29	Male	57	T4	N3	M0	39
30	Male	57	T4	N2	M0	3
31	Male	54	T3	N1	M0	33
32	Male	83	T4	N2	M0	21
33	Female	59	T3	N3	M0	45
34	Male	68	T2	N0	M0	4
35	Male	58	T2	N3	M1	60
36	Male	72	T3	N3	M0	15
37	Female	46	T2	N0	M0	17
38	Male	61	T3	N2	M0	10
39	Male	47	T3	N0	M0	10
40	Male	73	T3	N0	M0	30
41	Male	68	T4	N2	M0	38
42	Male	60	T2	N2	M0	38
43	Female	62	T3	N0	M0	42
44	Male	65	T2	N0	M0	50
45	Male	62	T3	N0	M0	48

46	Male	54	T2	N1	M0	58
47	Female	67	T3	N1	M0	32
48	Male	63	T4	N0	M0	39
49	Female	60	T1	N0	M0	2
50	Male	54	T2	N0	M0	31
51	Male	63	T3	N0	M0	36
52	Male	53	T3	N2	M0	58
53	Male	57	T2	N2	M0	31
54	Male	48	T3	N3	M0	23
55	Male	40	T1	N1	M0	40
56	Male	55	T2	N1	M0	30
57	Male	70	T3	N0	M0	16
58	Female	51	T2	N0	M0	11
59	Male	71	T3	N1	M0	26
60	Male	49	T2	N2	M0	42
61	Male	58	T3	N1	M0	18
62	Male	58	T2	N2	M0	47
63	Male	67	T3	N2	M0	39
64	Male	49	T4	N2	M0	35
65	Male	45	T1	N0	M0	12
66	Male	56	T2	N0	M0	9
67	Male	60	T3	N2	M0	13
68	Male	56	T1	N1	M0	38
69	Female	49	T3	N1	M0	11
70	Male	48	T2	N1	M0	1

Supplemental Table 4. Information of patients enrolled in the human breast cancer tissue microarray (HBreD136Su02, Outdo Biotech).

NO.	Sex	Age	Number of metastasis positive lymph nodes	T	N	M	AJCC stage	Survival (months)
1	Female	49	0	T2	N0	M0	2A	119
2	Female	55	22	T2	N3	M0	3C	119
3	Female	52	0	T2	N0	M0	2A	119
4	Female	44	3	T3	N1	M0	3A	22
5	Female	54	0	T1	N0	M0	1A	118
6	Female	61	1	T1	N1	M0	2A	118
7	Female	66	0	T2	N0	M0	2A	117
8	Female	73	0	T1	N0	M0	1A	101
9	Female	50	0	T1	N0	M0	1A	117
10	Female	69	1	T2	N1	M0	2B	117
11	Female	72	5	T1	N2	M0	3A	117
12	Female	55	14	T1	N3	M0	3C	34
13	Female	59	0	T1	N0	M0	1A	116
14	Female	48	5	T2	N2	M0	3A	11
15	Female	47	2	T2	N1	M0	2B	56
16	Female	46	4	T2	N2	M0	3A	112
17	Female	45	2	T1	N1	M0	2A	112
18	Female	52	1	T1	N1	M0	2A	112
19	Female	45	3	T2	N1	M0	2B	8
20	Female	56	0	T2	N0	M0	2A	110
21	Female	71	5	T2	N2	M0	3A	40
22	Female	41	9	T2	N2	M0	3A	64
23	Female	67	2	T2	N1	M0	2B	103
24	Female	72	2	T2	N1	M0	2B	107
25	Female	74	0	T2	N0	M0	2A	107
26	Female	57	2	T1	N1	M0	2A	107
27	Female	42	0	T2	N0	M0	2A	37
28	Female	63	0	T2	N0	M0	2A	105
29	Female	53	1	T2	N1	M0	2B	103
30	Female	61	0	T1	N0	M0	1A	101
31	Female	49	3	T2	N1	M0	2B	101
32	Female	49	12	T2	N3	M0	3C	16
33	Female	71	0	T1	N0	M0	1A	97
34	Female	60	0	T1	N0	M0	1A	96
35	Female	47	0	T2	N0	M0	2A	96
36	Female	64	0	T1	N0	M0	1A	96
37	Female	54	7	T1	N2	M0	3A	27
38	Female	51	0	T2	N0	M0	2A	92
39	Female	52	3	T1	N1	M0	2A	91
40	Female	60	2	T2	N1	M0	2B	91
41	Female	69	13	T3	N3	M0	3C	88
42	Female	72	0	T3	N0	M0	2B	87
43	Female	49	3	T1	N1	M0	2A	86
44	Female	88	0	T2	N0	M0	2A	39
45	Female	51	0	T1	N0	M0	1A	86
46	Female	57	1	T2	N1	M0	2B	28
47	Female	69	0	T2	N0	M0	2A	83
48	Female	82	0	T3	N0	M0	2B	83
49	Female	44	9	T3	N2	M0	3A	83
50	Female	54	1	T3	N1	M0	3A	79
51	Female	47	18	T2	N3	M0	3C	30
52	Female	58	0	T2	N0	M0	2A	77
53	Female	44	1	T2	N1	M0	2B	77
54	Female	48	2	T2	N1	M0	2B	67
55	Female	74	0	T1	N0	M0	1A	74
56	Female	53	0	T2	N0	M0	2A	74
57	Female	76	0	T2	N0	M0	2A	73
58	Female	65	0	T1	N0	M0	1A	72
59	Female	54	4	T3	N2	M0	3A	35
60	Female	84	0	T1	N0	M0	1A	49
61	Female	61	2	T2	N1	M0	2B	68
62	Female	57	0	T1	N0	M0	1A	68

63	Female	64	0	T1	N0	M0	1A	115
64	Female	57	2	T1	N1	M0	2A	115
65	Female	42	2	T2	N1	M0	2B	17
66	Female	76	1	T1	N1	M0	2A	112
67	Female	60	9	T2	N2	M0	3A	112
68	Female	75	0	T2	N0	M0	2A	108
69	Female	48	6	T2	N2	M0	3A	108
70	Female	42	1	T2	N1	M0	2B	106
71	Female	44	9	T3	N2	M0	3A	106
72	Female	48	3	T2	N1	M0	2B	105
73	Female	51	12	T2	N3	M0	3C	105
74	Female	54	4	T1	N2	M0	3A	103
75	Female	84	3	T2	N1	M0	2B	102
76	Female	52	6	T2	N2	M0	3A	102
77	Female	72	0	T2	N0	M0	2A	102
78	Female	49	0	T2	N0	M0	2A	101
79	Female	70	4	T2	N2	M0	3A	99
80	Female	58	0	T1	N0	M0	1A	70
81	Female	71	0	T2	N0	M0	2A	95
82	Female	68	3	T2	N1	M0	2B	95
83	Female	52	0	T1	N0	M0	1A	39
84	Female	37	1	T1	N1	M0	2A	95
85	Female	68	0	T2	N0	M0	2A	95
86	Female	74	0	T2	N0	M0	2A	42
87	Female	58	0	T2	N0	M0	2A	53
88	Female	51	1	T2	N1	M0	2B	94
89	Female	55	4	T2	N2	M0	3A	91
90	Female	71	0	T2	N0	M0	2A	88
91	Female	50	0	T3	N0	M0	2B	58
92	Female	80	3	T3	N1	M0	3A	39
93	Female	57	1	T2	N1	M0	2B	88
94	Female	62	23	T2	N3	M0	3C	87
95	Female	58	2	T2	N1	M0	2B	60
96	Female	86	0	T2	N0	M0	2A	40
97	Female	78	13	T3	N3	M0	3C	4
98	Female	72	0	T2	N0	M0	2A	83
99	Female	59	0	T2	N0	M0	2A	82
100	Female	59	11	T2	N3	M0	3C	82
101	Female	71	3	T2	N1	M0	2B	21
102	Female	87	0	T2	N0	M0	2A	34
103	Female	69	11	T2	N3	M0	3C	2
104	Female	49	5	T2	N2	M0	3A	27
105	Female	75	1	T3	N1	M0	3A	30
106	Female	43	0	T2	N0	M0	2A	78
107	Female	55	2	T2	N1	M0	2B	78
108	Female	60	19	T2	N3	M0	3C	33
109	Female	67	7	T2	N2	M0	3A	77
110	Female	49	0	T2	N0	M0	2A	76
111	Female	45	4	T2	N2	M0	3A	54
112	Female	62	0	T2	N0	M0	2A	74
113	Female	53	0	T2	N0	M0	2A	74
114	Female	88	4	T2	N2	M0	3A	73
115	Female	70	0	T2	N0	M0	2A	73
116	Female	49	0	T2	N0	M0	2A	72
117	Female	84	0	T1	N0	M0	1A	36
118	Female	74	0	T2	N0	M0	2A	71
119	Female	40	0	T2	N0	M0	2A	69
120	Female	37	0	T1	N0	M0	1A	69
121	Female	55	2	T1	N1	M0	2A	69
122	Female	56	0	T2	N0	M0	2A	68
123	Female	46	11	T2	N3	M0	3C	68
124	Female	64	0	T2	N0	M0	2A	67

Supplemental Table 5. Antibodies used in the study.

REAGENT or RESOURCE	SOURCE	IDENTIFIFER
Rabbit anti-SPEN	Novus	Cat# NBP1-82952
Rabbit anti-SPEN	Novus	Cat# NB100-58799
Rabbit anti-ERG	Abcam	Cat# ab92513
Rat anti-CD31	Biolegend	Cat# 102502
Goat anti-CD31	R&D	Cat# AF3628
Rabbit anti-α-SMA	Abcam	Cat# ab124964
Rabbit anti-Ki67	Abcam	Cat# ab15580
Rabbit anti-NG2	Millipore	Cat# AB5320
Rabbit anti-laminin	Sigma	Cat# L9393
Rabbit anti-p53	Proteintech	Cat# 10442-1-AP
Rabbit anti-p21 (human)	Proteintech	Cat# 10355-1-AP
Rabbit anti-p21 (mouse)	Proteintech	Cat# 28248-1-AP
Mouse anti-β-actin	Proteintech	Cat# 66009-1-Ig
Rabbit anti-GADD45A	CST	Cat# 4632
Rabbit anti-Hes1	Abcam	Cat# ab71559
Rabbit anti-ETS1	CST	Cat# 14069
Rabbit anti-VEGFR2	CST	Cat# 9698
Rabbit anti-Angpt2	Abcam	Cat# ab8452
Mouse anti-NPM1	Invitrogen	Cat# 32-5200
Mouse anti-RPA40	Santa Cruz	Cat# sc-374443
Mouse anti-FBL	Abcam	Cat# ab4566
Goat anti-NPM1	Abcam	Cat# ab31319
Rabbit anti-NPM1	Abcam	Cat# ab183340
Rabbit anti-CTCF	Millipore	Cat# 07-729
Mouse anti-UBF	Santa Cruz	Cat# sc-13125
Mouse anti-RPA194	Santa Cruz	Cat# sc-48385
Rabbit anti-H3K4me2	Millipore	Cat# 07-030
Rabbit anti-H2A.Z	Abcam	Cat# ab4174
Rabbit anti-H3ac	Millipore	Cat# 06-599
Rabbit anti-H3K27me3	Millipore	Cat# 07-449
Rabbit anti-H4K20me3	Millipore	Cat# 07-463
Rabbit anti-MDM2	CST	Cat# 86934
Rabbit anti-CTGF	Proteintech	Cat# 25474-1-AP
Rabbit anti-PIN1	Proteintech	Cat# 10495-1-AP
Rabbit anti-VEGFR3	Proteintech	Cat# 20712-1-AP
Rabbit anti-ZO-1	Proteintech	Cat# 21773-1-AP
Goat anti-VE-cadherin	R&D	Cat# AF1002
Rat anti-HSPG2	Invitrogen	Cat# MA5-14641
Rabbit anti-RPL5	Abcam	Cat# ab86863
Rabbit anti-RPL11	Proteintech	Cat# 16277-1-AP
Mouse anti-phospho-p53(Ser15)	CST	Cat# 9286S
Rabbit anti-phospho-p53(Ser20)	Abmart	Cat# TP56396S
Rabbit anti- phospho-p53(Thr18)	Abmart	Cat# TA2377S
APC anti-mouse CD45R/B220	Biolegend	Cat# 103211
FITC rat anti-mouse CD8α	BD	Cat# 553030
PE rat anti-mouse CD4	BD	Cat# 553048
APC rat anti-mouse CD3	Biolegend	Cat# 100236
Rabbit anti-LaminA/C	CST	Cat# 2032
HRP mouse anti-rabbit IgG (Light-Chain Specific)	CST	Cat# 93702
HRP rabbit anti-mouse IgG (Light-Chain Specific)	CST	Cat# 58802
HRP anti-rabbit IgG(H+L)	CST	Cat# 7074
HRP anti-mouse IgG(H+L)	CST	Cat# 7076
Alexa Fluor 488 donkey anti-rabbit IgG (H+L)	Invitrogen	Cat# A-21206
Alexa Fluor 594 donkey anti-rabbit IgG (H+L)	Invitrogen	Cat# A-21207
Alexa Fluor 488 donkey anti-rat IgG (H+L)	Invitrogen	Cat# A-21208
Alexa Fluor 594 donkey anti-rat IgG (H+L)	Invitrogen	Cat# A-21209
Alexa Fluor 647 goat anti-rabbit IgG (H+L)	Invitrogen	Cat# A-21245
Alexa Fluor 647 goat anti-rat IgG (H+L)	Invitrogen	Cat# A-21247
Alexa Fluor 594 donkey anti-goat IgG (H+L)	Invitrogen	Cat# A-11058
Alexa Fluor 594 donkey anti-mouse IgG (H+L)	Invitrogen	Cat# A-21203
Alexa Fluor 488 donkey anti-mouse IgG (H+L)	Invitrogen	Cat# A-21202
Alexa Fluor 647 donkey anti-mouse IgG (H+L)	Invitrogen	Cat# A-31571
Alexa Fluor 647 donkey anti-goat IgG (H+L)	Invitrogen	Cat# A-21447
Alexa Fluor 488 rabbit anti-ERG	Abcam	Cat# ab196374
Normal Rabbit IgG	CST	Cat# 3900
Normal Mouse IgG	Millipore	Cat# 12-371

Supplemental Table 6. List of primers.

Primers	Sequence	Application
CreN1	CCGGTCGATGCAACGAGTGATGAGG	PCR
CreN2	GCCTCCAGCTTGCATGATCTCCGG	PCR
RBPj R3	GTTCTTAACCTGTTGGTCGGAACC	PCR
RBPj R4	GCTTGAGGCTTGATGTTCTGTATTGC	PCR
RBPj PGKD	ACCGGTGGATGTGGAATGTGT	PCR
SPEN C (F)	CGCCCTCAGGCCTCCACCACTTGCG	PCR
SPEN W (R1)	GCACAGTGCACAGATACTCACGC	PCR
SPEN KK (R2)	TGGAGATGGAAAGAAGACAAAGG	PCR
p53 flox F	GAGCATGGAAGTAAGACCCCTTCT	PCR
p53 flox R	GACAGGGTTTCTCTATGTAGCCCT	PCR
Mouse β-actin F	GGCTGTATTCCCCTCCATCG	RT-qPCR
Mouse β-actin R	CCAGTTGGTAACAATGCCATGT	RT-qPCR
Human β-actin F	TGGCACCCAGCACAATGAA	RT-qPCR
Human β-actin R	CTAAGTCATAGTCCGCCTAGAAGCA	RT-qPCR
Mouse SPEN F	GCTGAGCTACTCGGGACAGAA	RT-qPCR
Mouse SPEN R	GATCTGGCTGATCTTAGCACTGA	RT-qPCR
Human SPEN F	CAAAGGGCGCCAGAAAACAA	RT-qPCR
Human SPEN R	CTTCGGGGTGCTGTACTGTT	RT-qPCR
Human p21 F	AGGTGGACCTGGAGACTCTCAG	RT-qPCR
Human p21 R	TCCTCTTGGAGAAGATCAGCCG	RT-qPCR
Mouse p21 F	CCTGGTGATGTCCGACCTG	RT-qPCR
Mouse p21 R	CCATGAGCGCATCGCAATC	RT-qPCR
Mouse p53 F	TATTCTGCCAGCTGGCGAAGACGTGC	RT-qPCR
Mouse p53 R	TGGTGGTATACTCAGAGCCGGCCTCG	RT-qPCR
Human p53 F	CCTCAGCATCTTATCCGAGTGG	RT-qPCR
Human p53 R	TGGATGGTGGTACAGTCAGAGC	RT-qPCR
Human MDM2 F	TGTTTGGCGTGCCAAGCTTCTC	RT-qPCR
Human MDM2 R	CACAGATGTACCTGAGTCCGATG	RT-qPCR
Human GADD45A F	CTGGAGGAAGTGCTCAGCAAAG	RT-qPCR
Human GADD45A R	AGAGCCACATCTCTGTCGTCGT	RT-qPCR
Human GADD45B F	GCCAGGATCGCCTCACAGTGG	RT-qPCR
Human GADD45B R	GGATTTGCAGGGCGATGTCATC	RT-qPCR
Mouse Hes-1 F	TCAACACGACACCGGACAAAC	RT-qPCR
Mouse Hes-1 R	ATGCCGGGAGCTATCTTTCTT	RT-qPCR
Mouse Hey-1 F	CCGACGAGACCGAATCAATAAC	RT-qPCR
Mouse Hey-1 R	TCAGGTGATCCACAGTCATCTG	RT-qPCR
Human Hes-1 F	GGAAATGACAGTGAAGCACCTCC	RT-qPCR
Human Hes-1 R	GAAGCGGGTCACCTCGTTCATG	RT-qPCR
Human Hey-1 F	TGTCTGAGCTGAGAAGGCTGGT	RT-qPCR
Human Hey-1 R	TTCAGGTGATCCACGGTCATCTG	RT-qPCR
Mouse HSPG2 F	CATTCAGGTGGTCGTCCTCTCA	RT-qPCR
Mouse HSPG2 R	AGGTCAAGCGTCTGTCCTTCAG	RT-qPCR
Mouse CTGF F	TGCGAAGCTGACCTGGAGGAAA	RT-qPCR
Mouse CTGF R	CCGCAGAACTTAGCCCTGTATG	RT-qPCR
Human HSPG2 F	TCAGGCGAGTATGTGTGCCATG	RT-qPCR
Human HSPG2 R	GATGAAGACTCGATCCTGACAGG	RT-qPCR
Human CTGF F	CTTGCGAAGCTGACCTGGAAGA	RT-qPCR
Human CTGF R	CCGTCGGTACATACTCCACAGA	RT-qPCR
Human ETS1 F	GAGTCAACCCAGCCTATCCAGA	RT-qPCR
Human ETS1 R	GAGCGTCTGATAGGACTCTGTG	RT-qPCR
Mouse ETS1 F	CCAGAATCCTGTTACACCTCGG	RT-qPCR
Mouse ETS1 R	CAGCGTCTGATAGGACTCTGTG	RT-qPCR
Human ANGPT2 F	ATTCAGCGACGTGAGGATGGCA	RT-qPCR
Human ANGPT2 R	GCACATAGCGTTGCTGATTAGTC	RT-qPCR
Mouse ANGPT2 F	AACTCGCTCCTTCAGAAGCAGC	RT-qPCR
Mouse ANGPT2 R	TTCCGCACAGTCTCTGAAGGTG	RT-qPCR
Human VEGFR2 F	GGAACCTCACTATCCGCAGAGT	RT-qPCR
Human VEGFR2 R	CCAAGTTCGTCTTTTCCTGGGC	RT-qPCR
Mouse VEGFR2 F	CGAGACCATTGAAGTGACTTGCC	RT-qPCR
Mouse VEGFR2 R	TTCCTCACCCTGCGGATAGTCA	RT-qPCR
Human VEGFR3 F	TGCGAATACCTGTCCTACGATGC	RT-qPCR
Human VEGFR3 R	CTTGTGGATGCCGAAAGCGGAG	RT-qPCR
Mouse VEGFR3 F	AGACTGGAAGGAGGTGACCACT	RT-qPCR
Mouse VEGFR3 R	CTGACACATTGGCATCCTGGATC	RT-qPCR
Human RPL5 F	CCAAATACAGGATGATAGTTCGTG	RT-qPCR
Human RPL5 R	TTGGCAGTTCGTGTGCATACGC	RT-qPCR
Human RPL11 F	AGAGTGGAGACAGACTGACGCG	RT-qPCR
Human RPL11 R	CGGATGCCAAAGGATCTGACAG	RT-qPCR
Human RPL23 F	ATCAAGGGACGGCTGAACAGAC	RT-qPCR
Human RPL23 R	GTCGAATGACCACTGCTGGATG	RT-qPCR
Human pre-rRNA F	GCCTTCTCTAGCGATCTGAGAG	RT-qPCR
Human pre-rRNA R	CCATAACGGAGGCAGAGACA	RT-qPCR
Human 18S rRNA F	CGCCGCGCTCTACCTTACCTA	RT-qPCR
Human 18S rRNA R	TAGGAGAGGAGCGAGCGACCA	RT-qPCR
Human 28S rRNA F	CTCCGAGACGCGACCTCAGAT	RT-qPCR
Human 28S rRNA R	CGGGTCTTCCGTACGCCACAT	RT-qPCR

Human 5.8S rRNA F	GAGGCAACCCCCTCTCCTCTT	RT-qPCR
Human 5.8S rRNA R	GAGCCGAGTGATCCACCGCTA	RT-qPCR
human 5S rRNA F	GGCCATACCACCCTGAACGC	RT-qPCR
human 5S rRNA R	CAGCACCCGGTATTCCCAGG	RT-qPCR
Mouse RPL5 F	GCGCTACCTAATGGAGGAAGATG	RT-qPCR
Mouse RPL5 R	CTCTCGGATAGCAGCATGAGCT	RT-qPCR
Mouse RPL11 F	GAGAGCGGAGACAGACTGACC	RT-qPCR
Mouse RPL11 R	GGATGCCAAAGGACCTGACAGT	RT-qPCR
Mouse RPL23 F	ACGGCTGAACAGACTTCCTGCT	RT-qPCR
Mouse RPL23 R	CGTTGTCGAATTACCACTGCTGG	RT-qPCR
Mouse pre-rRNA F	CTCTTGTTCTGTGTCTGCC	RT-qPCR
Mouse pre-rRNA R	GCCCGCTGGCAGAACGAGAAG	RT-qPCR
Mouse 18S rRNA F	GTAACCCGTTGAACCCCATT	RT-qPCR
Mouse 18S rRNA R	CCATCCAATCGGTAGTAGCG	RT-qPCR
Mouse 28S rRNA F	AAGCGGGTGGTAAACTCCATCTAAG	RT-qPCR
Mouse 28S rRNA R	CCACCCGTTTACCTCTTAACGGTTTC	RT-qPCR
Mouse 5.8S rRNA F	GACTCTTAGCGGTGGATCACTCGGC	RT-qPCR
Mouse 5.8S rRNA R	CGCAAGTGCGTTCGAAGTGTCGATG	RT-qPCR
Human CTCF F	TGCGGAAAGTGAACCCAT	RT-qPCR
Human CTCF R	TTTTGGCTGGTGGCTGAT	RT-qPCR
H42.1 F	GCTTCTCGACTCACGGTTTC	CHIP-qPCR
H42.1 R	CCGAGAGCACGATCTCAAA	CHIP-qPCR
H42.9 F	CCCGGGGGAGGTATATCTTT	CHIP-qPCR
H42.9 R	CCAACCTCTCCGACGACA	CHIP-qPCR
IGS-18 F	GTTGACGTACAGGGTGGACTG	ss-RT-qPCR
IGS-18 R	GGAAGTTGTCTTCACGCCTGA	ss-RT-qPCR
IGS-22 F	CAGTGGCTCACGTCTGTCAT	ss-RT-qPCR
IGS-22 R	CGCCTGACTCCATTTCGTAT	ss-RT-qPCR
IGS-28 F	CCTTCCACGAGAGTGAGAAG	ss-RT-qPCR
IGS-28 R	GACCTCCCGAAATCGTACAC	ss-RT-qPCR
Human 7SK RNA F	AGGACCGGTCTTCGGTCAA	ss-RT-qPCR
Human 7SK RNA R	TCATTTGGATGTGTCTGCAGTCT	ss-RT-qPCR
Human PAPAS (-49/-30) F	GGTATATCTTTCGCTCCGAG	ss-RT-qPCR
Human PAPAS (+13/+32) R	GACGACAGGTCGCCAGAGGA	ss-RT-qPCR
Human pRNA (-194/-169) F	TGTGTCCTTGGGTTGACCAGAGGGAC	ss-RT-qPCR
Human pRNA (-1/-25) R	ATATAACCCGGCGGCCCAAAATTGC	ss-RT-qPCR
Mouse pRNA (-131/-106) F	TTATGGGGTCATTTTTGGGCCACCTC	ss-RT-qPCR
Mouse pRNA (-1/-26) R	ACCTATCTCCAGGTCCAATAGGAACA	ss-RT-qPCR
Mouse 7SK RNA F	TCAAGGGTATACGAGTAGCTGCGCTC	ss-RT-qPCR
Mouse 7SK RNA R	GATGTGTCTGGAGTCTTGGAAGCTTG	ss-RT-qPCR

Supplemental Video 1 and 2. Time-lapse microscopy of the HUVECs transduced with NC or SPENi lentivirus.

HUVECs were transduced with NC (Supplemental Video 1) or SPENi (Supplemental Video 2) lentivirus expressing EGFP and recorded with a living cell imaging workstation under a fluorescence microscope at 5-min intervals.
